# The adjuvant GLA-SE promotes human Tfh cell expansion and emergence of public TCRβ clonotypes

**DOI:** 10.1084/jem.20190301

**Published:** 2019-06-07

**Authors:** Danika L. Hill, Wim Pierson, Daniel J. Bolland, Catherine Mkindi, Edward J. Carr, Jiong Wang, Sophie Houard, Steven W. Wingett, Regine Audran, Elizabeth F. Wallin, Said A. Jongo, Kassim Kamaka, Martin Zand, Francois Spertini, Claudia Daubenberger, Anne E. Corcoran, Michelle A. Linterman

**Affiliations:** 1 Laboratory of Lymphocyte Signalling and Development, Babraham Institute, Cambridge, UK; 2 Ifakara Health Institute, Bagamoyo, Tanzania; 3 Department of Medicine, University of Cambridge, Cambridge, UK; 4 Division of Nephrology, Department of Medicine and Clinical and Translational Science Institute, University of Rochester Medical Center, Rochester, NY; 5 European Vaccine Initiative, Heidelberg, Germany; 6 Babraham Bioinformatics Facility, Babraham Institute, Cambridge, UK; 7 Division of Immunology and Allergy, Centre Hospitalier Universitaire Vaudois, Lausanne, Switzerland; 8 Renal Department, Lister Hospital, Stevenage, UK; 9 Swiss Tropical and Public Health Institute, Basel, Switzerland; 10 University of Basel, Switzerland

## Abstract

The generation of protective humoral immunity after vaccination relies on the productive interaction between antigen-specific B cells and T follicular helper (Tfh) cells. Despite the central role of Tfh cells in vaccine responses, there is currently no validated way to enhance their differentiation in humans. From paired human lymph node and blood samples, we identify a population of circulating Tfh cells that are transcriptionally and clonally similar to germinal center Tfh cells. In a clinical trial of vaccine formulations, circulating Tfh cells were expanded in Tanzanian volunteers when an experimental malaria vaccine was adjuvanted in GLA-SE but not when formulated in Alum. The GLA-SE–formulated peptide was associated with an increase in the extrafollicular antibody response, long-lived antibody production, and the emergence of public TCRβ clonotypes in circulating Tfh cells. We demonstrate that altering vaccine adjuvants is a rational approach for enhancing Tfh cells in humans, thereby supporting the long-lived humoral immunity that is required for effective vaccines.

## Abstract (precis)

A rational strategy to achieve optimal vaccine responses is to potentiate Tfh cells and the germinal center response. This work shows the adjuvant GLA-SE enhances circulating Tfh cells and enduring antibody responses to a malaria vaccine in Tanzanian adults.

## Graphical Abstract

**Figure:**
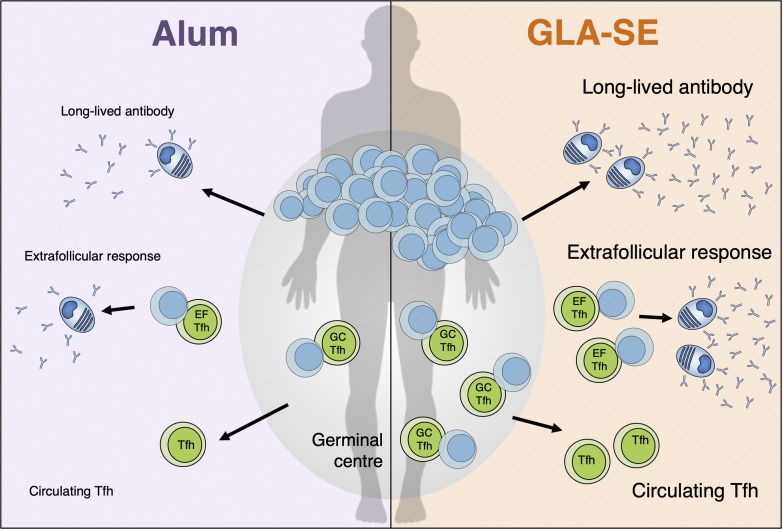

## Introduction

Vaccination is one of the most powerful interventions for reducing the disability and death caused by infectious disease worldwide (Andre et al., 2008). Despite its success, there are still numerous pathogens that are not controlled by current vaccination strategies, including HIV and *Plasmodium spp.*, the parasites that cause malaria (Burton et al., 2012; Delany et al., 2014). The majority of effective vaccines work by stimulating the production of antibodies that bind the surface of the pathogen to either block the pathogen’s ability to establish an infection or recruit and stimulate other immune cells, resulting in the pathogen’s destruction. Antibody production after vaccination can occur via two cellular pathways, which although separated in time and anatomical space, are both induced after vaccination with a protein antigen. The first wave of antibody production comes from the extrafollicular plasmablast response, in which short-lived antibody-secreting cells (ASCs) produce immunoglobulin for a few days (MacLennan et al., 2003). The second wave of ASCs comes from the germinal center (GC) response. The GC is a specialized microenvironment that forms in secondary lymphoid tissues after immunization, where antigen-activated B cells clonally expand within the B cell follicle and undergo somatic hypermutation (SHM) of their immunoglobulin loci. The process of SHM, followed by the affinity-based selection of GC B cells, results in the emergence of long-lived, high-affinity, antibody-secreting plasma cells and memory B cells that are able to provide protection against subsequent infection (Vinuesa et al., 2016). The GC response is absolutely dependent on a specialized subset of CD4^+^ T cells, T follicular helper (Tfh) cells, which provide growth and differentiation signals to GC B cells. Tfh cells mediate the positive selection of B cell clones in the GC and thereby determine which B cells exit the GC as plasma cells or memory B cells (Vinuesa et al., 2016). Because Tfh cells are key determinants of the long-lived humoral immunity that arises from the GC, they represent an exciting therapeutic target through which vaccine strategies could be improved (Linterman and Hill, 2016). This is particularly pertinent for malaria vaccine development, as clinical trials indicate that the development of long-lived antibody responses, in particular for the preerythrocytic and blood stages of infection, are essential for sustained protective immunity (Cockburn and Seder, 2018).

Adjuvants are an attractive way to improve vaccine responses in humans. This is reflected in the licensing of four new adjuvants in recent years: MF59, AS01, AS02, and AS04 (Garçon and Di Pasquale, 2017). Nevertheless, the number of adjuvants that are in use in current vaccines is still very limited, largely unchanged since the 1940s, as is our understanding of how adjuvants boost a specific cellular immune response in humans. While studies in animals indicate that adjuvants are a valid way to boost the GC and Tfh cell response (Aloulou et al., 2016; Desbien et al., 2016; Liang et al., 2017), translational work is needed to determine if using novel adjuvants can boost these responses in humans. Despite Tfh cells being central for long-term humoral immunity, most human vaccine studies have not included these cells in their analysis, rather focusing on cytokine-producing CD4^+^ T cells (Coler et al., 2015), an approach that does not accurately capture vaccine-reactive Tfh cells (Dan et al., 2016). The tendency to omit the analysis of Tfh cells in human vaccination studies may be due to difficulty in studying these cellular responses. Tfh cells are located in secondary lymphoid tissue, which is not easily sampled during vaccine trials. To circumvent this issue, a population of circulating Tfh-like (cTfh) cells that are found in the blood and phenotypically and functionally resemble lymphoid tissue Tfh cells can be used as a biomarker of ongoing Tfh cell responses (Simpson et al., 2010; Chevalier et al., 2011; Morita et al., 2011; Bentebibel et al., 2013; He et al., 2013; Locci et al., 2013). Here, we have used a seasonal influenza vaccination study and analyses of CXCR5^+^ Tfh cell populations from the blood and LNs of human donors to refine our understanding of cTfh cells as circulating biomarkers of bona fide Tfh cells resident in secondary lymphoid tissues. We then applied this knowledge to determine whether the combination adjuvant glucopyranosyl lipid adjuvant-stable emulsion (GLA-SE; developed by the Infectious Disease Research Institute, Seattle, WA) can augment cTfh cell responses. In a phase Ib malaria vaccine trial in Tanzania, we show that a GLA-SE–formulated vaccine is superior to one formulated with Alhydrogel (Alum). GLA-SE induced a greater extrafollicular antibody response and increased the formation of cTfh cells, including cTfh cells with transcriptional similarity and a clonal relatedness to LN GC-Tfh cells. While the different adjuvants did not induce differential gene expression profiles in cTfh cells, multiple GLA-SE–vaccinated individuals had cTfh cells expressing public TCRβ clonotypes, indicating that GLA-SE may support the recruitment of T cells bearing specific TCRs to the Tfh cell compartment or promote their subsequent expansion. This demonstrates that experimental vaccine adjuvants offer a viable strategy to enhance Tfh responses and long-lived humoral immunity in humans.

## Results

### cTfh cells clonally expand after vaccination

**Figure 1.**
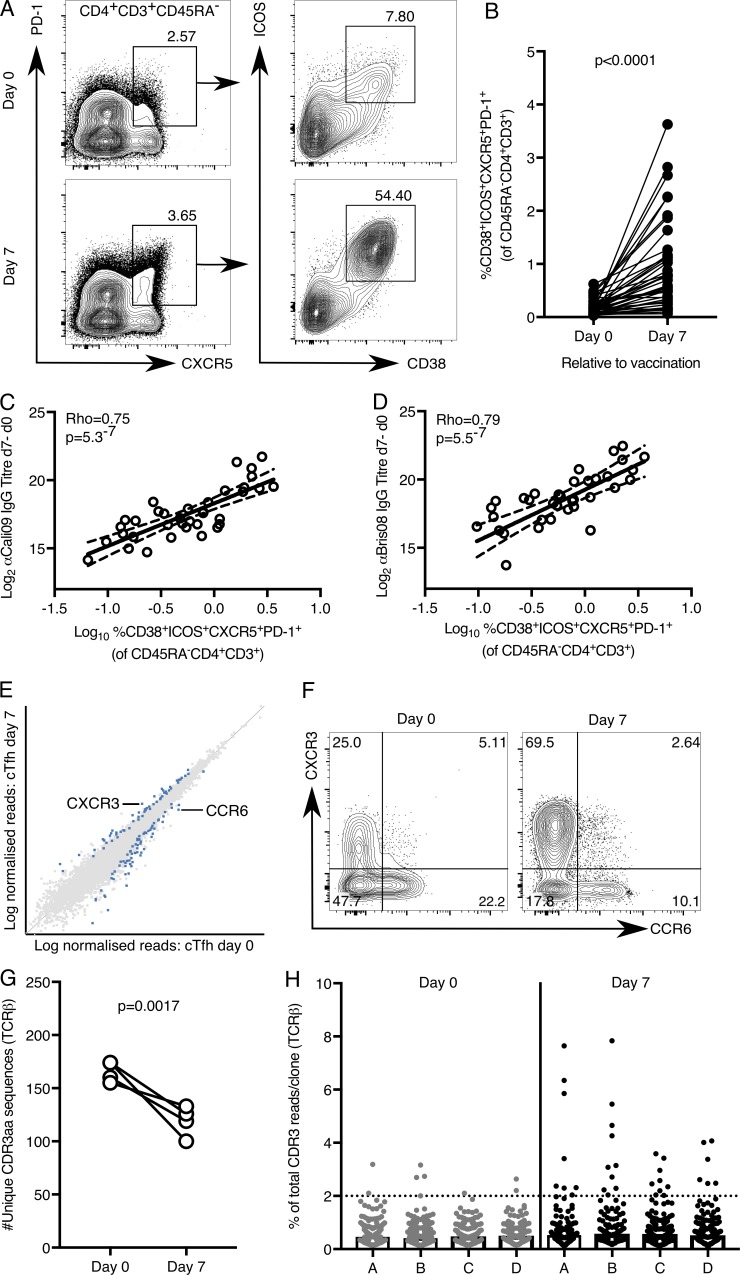
**ICOS and CD38 mark cTfh cells following seasonal influenza vaccination. (A and B)** Flow cytometric contour plots (A) and quantification (B) of the frequency of CD38^+^ICOS^+^CXCR5^+^PD-1^+^ cells among CD45RA^−^CD4^+^CD3^+^ cells in the peripheral blood of healthy UK donors at days 0 and 7 relative to seasonal influenza vaccination; *n* = 41. **(B)** Each symbol represents a volunteer; an individual donor is connected by a line at the two time points; *n* = 41. P < 0.0001; the P value was generated with a Wilcoxon signed-rank test. **(C and D)** Correlation of the frequency of CD38^+^ICOS^+^CXCR5^+^PD-1^+^ cTfh cells 7 d after vaccination with the change in antibody titer of anti-Cal09 IgG (C, an influenza A HA, P = 5.3 × 10^−7^, Rho = 0.75) and anti-Bris08 IgG (D, an influenza B HA, P = 5.5 × 10^−7^, Rho = 0.79) 7 d after vaccination. Statistical analysis by Spearman’s correlation (Rho = coefficient); *n* = 41. **(E)** Scatterplot of whole transcriptome RNA-sequencing data comparing the expression of all genes expressed in CD38^+^ICOS^+^CXCR5^+^PD-1^+^ cells before and 7 d following seasonal influenza vaccination; *n* = 4. Differentially expressed genes (DESeq2) are indicated in blue. **(F)** Flow cytometric contour plots of CXCR3 and CCR6 expression on ICOS^+^CXCR5^+^PD-1^+^ Tfh cells at the indicated time points relative to vaccination, a representative example of 36 individuals. **(G)** The number of unique TCRβ CDR3 amino acid sequences identified in RNA-sequencing libraries from CD38^+^ICOS^+^CXCR5^+^PD-1^+^ cTfh cells from four volunteers were analyzed at days 0 and 7 relative to vaccination; *n* = 4. P = 0.0017; the P value was generated with a paired Student’s *t* test. **(H)** Quantification of the percentage of total TCRβ CDR3 reads per unique clone: each symbol represents a unique CDR3 amino acid sequence. The dotted line indicates a frequency of 2%. In H, each letter on the x axis corresponds to a different volunteer. Data are from one seasonal influenza vaccination cohort.

The aim of our study was to determine whether the adjuvant GLA-SE promotes a cTfh cell response in humans. The location of the GC response, within secondary lymphoid tissues, is one of the major barriers to understanding how different vaccines, and their adjuvants, affect GC biology in humans. For this reason, circulating cells that can act as biomarkers of the GC response are an area of intense interest. In particular, it is well established that there is a population of blood CXCR5^+^CD4^+^ cells that expands after vaccination (Bentebibel et al., 2013; He et al., 2013; Locci et al., 2013) and that these cTfh cells phenotypically and functionally resemble lymphoid tissue Tfh cells (Linterman and Hill, 2016). There is considerable heterogeneity in the circulating CXCR5^+^CD4^+^ T cell compartment, with multiple subsets described within this population (Schmitt et al., 2014). Because of this heterogeneity, we first sought to identify the subpopulation of CXCR5^+^CD4^+^ T cells that were activated by vaccination to enable us to refine our analyses. Inducible costimulator (ICOS) and CD38 are receptors expressed on the cell surface of tonsillar Tfh cells but not CD45RA^−^ non-Tfh cells (Fig. S1, A–D). The frequency of ICOS^+^CD38^+^ tonsillar Tfh cells correlates with the percentage of GC B cells (Fig. S1 E), suggesting that cTfh cells expressing these markers may represent a circulating surrogate of activated lymphoid tissue Tfh cells. We first tested expression of these cell-surface receptors after seasonal influenza vaccination, a routine nonadjuvanted inoculation in which cTfh cell expansion has been well described (Bentebibel et al., 2013) and in which expression of ICOS and CD38 has been reported on cTfh cells (Bentebibel et al., 2013; Herati et al., 2017; Koutsakos et al., 2018). We observed an expansion of ICOS^+^CD38^+^CXCR5^+^PD-1^+^ cTfh cells in healthy UK volunteers (*n* = 41) 7 d after vaccination (Fig. 1, A and B), the peak of the cTfh cell response (Bentebibel et al., 2013; Carr et al., 2016). This expansion of ICOS^+^CD38^+^CXCR5^+^PD-1^+^ cTfh cells correlated positively with the increase in influenza-specific antibodies 7 and 42 d after vaccination (Fig. 1, C and D; and Fig. S1, F and G). In addition, using HLA-DR tetramers in a subset of the volunteers with the appropriate HLA genotype (Yang et al., 2013), we were able to identify hemagglutinin (HA)-specific ICOS^+^CD38^+^CXCR5^+^PD-1^+^ cTfh cells 7 d after vaccination (Fig. S2, A–C). These data indicate that ICOS^+^CD38^+^CXCR5^+^PD-1^+^ cTfh cells could be a good biomarker of lymphoid tissue Tfh cells that support humoral immunity.

To gain insight into how vaccines qualitatively alter Tfh cell responses, we performed RNA sequencing of 200 ICOS^+^CD38^+^CXCR5^+^PD-1^+^ cTfh cells immediately before and 7 d after influenza vaccination in four individuals. Differential gene expression analysis of these cTfh cells indicated up-regulation of *CXCR3* and down-regulation of *CCR6* transcripts in cTfh cells isolated 7 d after vaccination (Fig. 1 E). This difference in mRNA was also reflected at the protein level, with the proportion of CXCR3^+^ cTfh cells increasing after influenza vaccination and a reciprocal decrease in CCR6^+^ cTfh cells (Fig. 1 F). This is consistent with previous work demonstrating that influenza vaccination results in a Th1-skewing of the Tfh cell population (Bentebibel et al., 2013). Transcriptomic analysis also enabled an assessment of the TCR usage within cTfh cells. Assessment of the nucleotide sequence encoded by the CDR3 region of the TCRβ chain revealed ∼160 TCRβ clonotypes per person that were unique at the amino acid level before vaccination (Fig. 1 G), a number that is consistent with the 200 cells sequenced. After vaccination, the number of unique TCRβ clonotypes identified per person was reduced (Fig. 1 G), consistent with clonal expansion of cTfh after influenza vaccination as previously described (Herati et al., 2017). This reduction in diversity was driven by the expansion of a few clones: before vaccination, most TCRβ clonotypes represented <2% of the total CDR3 reads, whereas 7 d after vaccination, 6–10 unique CDR3 sequences per individual were present at higher frequencies (Fig. 1 H). This indicates that the cTfh cell response to influenza vaccination is dominated by the expansion of a small number of T cell clones. In summary, the combination of flow cytometric analysis and RNA sequencing identifies the ICOS^+^CD38^+^CXCR5^+^PD-1^+^ cTfh cell population as a biomarker of ongoing Tfh cell responses and provides a quantitative and qualitative way to assess the biology of these cells during human vaccine trials.

### A GLA-SE–formulated vaccine promotes long-lasting IgG responses in humans

**Figure 2.**
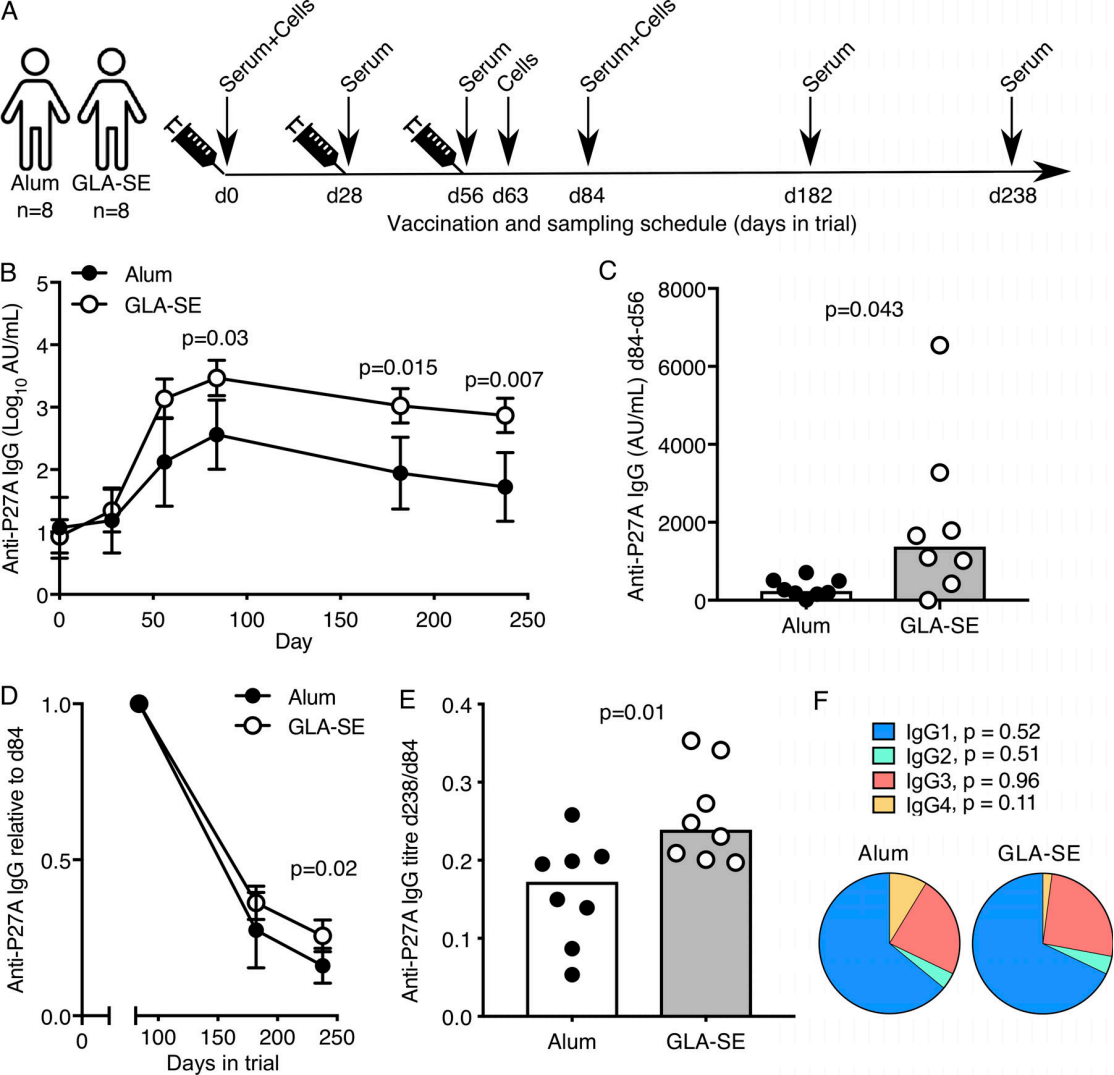
**The adjuvant GLA-SE enhances antibody production but not early ASC expansion. (A)** Vaccination and venepuncture schedule for the clinical trial. **(B)** Anti-P27A IgG antibody titers in volunteers vaccinated with 50 µg P27A peptide in either Alum (black) or GLA-SE (white); error bars represent the 95% confidence interval. Day 0, NS; day 28, NS; day 56, NS; day 84, P = 0.03; day 82, P = 0.015; day 238, P = 0.007. The P values were calculated using a two-way ANOVA with Sidak’s multiple-comparisons test; *n* = 8/group. Log10 anti-P27A titers are shown. **(C)** Change in anti-P27A IgG antibody titer 28 d after the third vaccination (d84); *n* = 8/group; P = 0.043. **(D and E)** Anti-P27A IgG titer normalized to the peak of the antibody response (d84) in volunteers vaccinated with 50 µg P27A peptide in either Alum (black) or GLA-SE (white); *n* = 8/group. In D, error bars represent the 95% confidence interval; P = 0.02 (D); P = 0.01 (E). The P values were calculated using a two-way ANOVA. **(F)** Pie chart of the total anti-P27A IgG pool at day 84 divided by immunoglobulin isotype; *n* = 8/group. IgG1, P = 0.52; IgG2, P = 0.51; IgG3, P = 0.96; IgG4, P = 0.11. In C and E, the height of the bar represents the median, and each symbol represents one individual; those who received Alum are shown in black, and those who received GLA-SE are in white. In C, E, and F, P values were calculated using a Mann-Whitney *U* test. Data are from one clinical trial.

The P27A antigen is a 104–amino acid peptide of the PFF0165c protein from the malaria-causing protozoan *Plasmodium falciparum*. Because seroreactivity to P27A is high in populations naturally exposed to malaria, and anti-P27A IgG can inhibit asexual blood stage parasite growth in vitro (Olugbile et al., 2009), P27A offers a rational vaccine target to prevent malaria disease. In a phase Ib clinical trial with adults from Tanzania, the P27A peptide (50 µg) was formulated with one of two adjuvants: the widely used Alum (Alhydrogel) or the experimental adjuvant GLA-SE (5 µg), a synthetic TLR4 agonist, enabling side-by-side comparison of vaccine-induced immunity (Steiner-Monard et al., 2019). All participants were malaria preexposed but had no clinical malaria episode recorded in the past 5 yr. The volunteers were given three doses of either vaccine, each 1 mo apart, with blood samples taken for serology at multiple time points over a 34-wk follow-up period (Steiner-Monard et al., 2019; sampling schedule in Fig. 2 A). In each participant, we detected low levels of anti-P27A antibodies before vaccination, which was not different between adjuvant groups, indicating that responses to the P27A vaccination involve reactivation of P27A-specific memory lymphocytes (Fig. 2 B). It is important to study vaccine responses in cohorts with preexposure, as only a vaccine that successfully induces protective immunity in individuals living in malaria-endemic regions will limit malarial disease burden. Individuals with preexposure have been shown to mount poorer humoral responses in experimental malaria vaccine trials than unexposed individuals, including to the P27A peptide (Steiner-Monard et al., 2019). After vaccination, P27A induced a higher anti-P27A IgG response when formulated in GLA-SE compared with Alum from day 63 through day 238 (Fig. 2 B). In contrast to Alum, a significant further increase in serum anti-P27A IgG titers was observed 28 d after the third vaccination in the GLA-SE group (Fig. 2 C). This is the peak of the antibody response in this study, and a time when vaccine-specific antibodies are most likely derived from both extrafollicular and GC-derived ASCs. To determine whether the adjuvant has an impact on the longevity of the antibody response, we calculated the proportion of peak (day 84, 28 d after the final vaccination) anti-P27A IgG antibodies that remain at later time points (Fig. 2 D). 6 mo after the final vaccination, individuals vaccinated with GLA-SE–formulated antigen had ∼25% of peak antibody titers remaining compared with ∼18% in the Alum-treated group (Fig. 2 E). There was no significant difference in the subclass of the IgG produced, with IgG1 dominating the response in both groups (Fig. 2 F). Together, these indicate that the adjuvant GLA-SE increases both the magnitude and longevity of the anti-P27A antibody response compared with Alum.

### GLA-SE drives higher frequencies of cTfh cells compared with Alum

**Figure 3.**
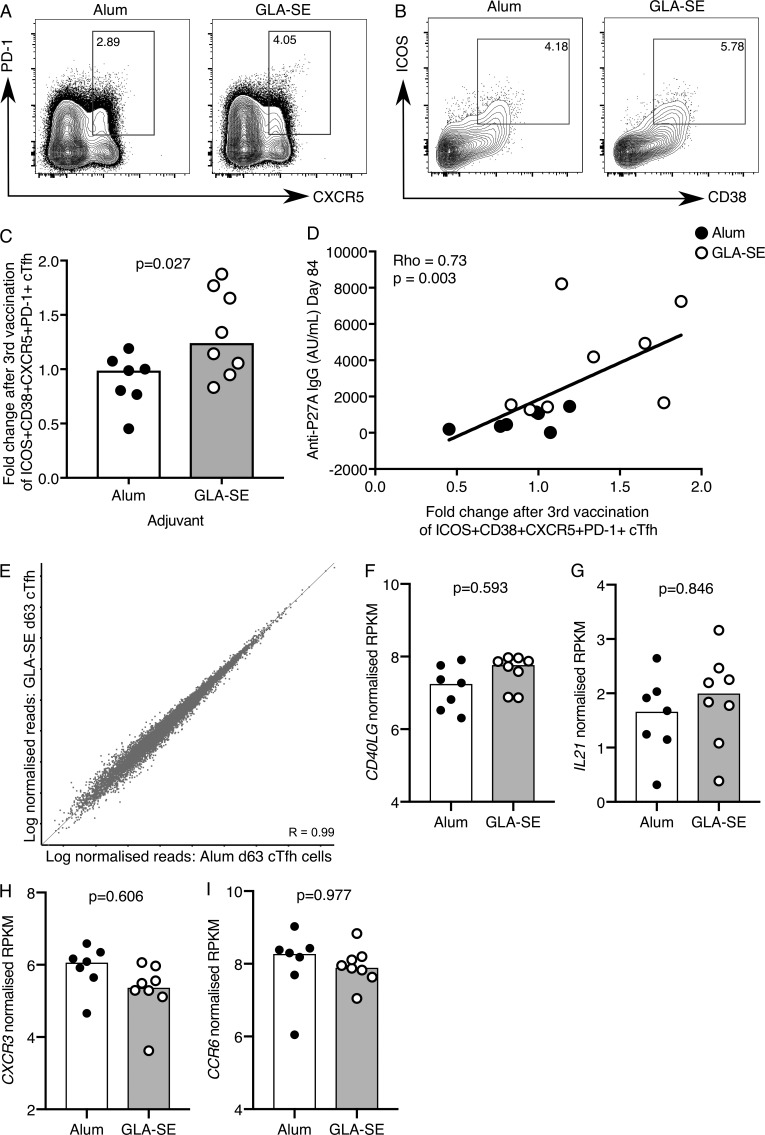
**A GLA-SE adjuvanted vaccine promotes cTfh expansion. (A and B)** Flow cytometric contour plots of (A) PD-1 and CXCR5 on total CD45RA^−^CD4^+^CD3^+^ cells and (B) ICOS and CD38 on the population gated in A on peripheral blood cells from individuals 7 d after the third P27A vaccination. Alum group, *n* = 7; GLA-SE group, *n* = 8. **(C)** Fold change of CD38^+^ICOS^+^CXCR5^+^PD-1^+^ cTfh cells 7 d after the third P27A vaccination (frequency of cTfh cells at day 63/baseline). Alum group, *n* = 7; GLA-SE, group *n* = 8. P = 0.027. The P value is from an unpaired Student’s *t* test. **(D)** Correlation of anti-P27A IgG antibody titer 28 d after the third vaccination against fold change in CD38^+^ICOS^+^CXCR5^+^PD-1^+^ cTfh cells 7 d after the third vaccination. Rho = 0.73; P = 0.003 by Spearman’s correlation (Rho = coefficient); *n* = 15. **(E)** Scatterplot of RNA-sequencing data comparing all genes expressed in CD38^+^ICOS^+^CXCR5^+^PD-1^+^ cTfh cells 7 d after the third P27A vaccination in either Alum- (x axis) or GLA-SE– (y axis) vaccinated groups. Alum group, *n* = 7; GLA-SE group, *n* = 8. **(F–I)** Bar plots showing log-normalized reads from RNA-sequencing data of *CD40LG*. P = 0.593 (F), *IL21* P = 0.846 (G), *CXCR3* P = 0.606 (H), and *CCR6* P = 0.977 (I) for transcripts in ICOS in CD38^+^ICOS^+^CXCR5^+^PD-1^+^ cTfh cells 7 d after the third P27A vaccination; Alum group, *n* = 7; GLA-SE group, *n* = 8. P values were calculated using DESeq2 with a false discovery rate correction. Each symbol represents one individual; those who received Alum are shown in black, and those who received GLA-SE are in white. Data are from one clinical trial.

Tfh cells regulate GC size and are essential for the emergence of long-lived plasma cells and memory B cells from the GC; therefore, boosting Tfh cell number is a rational strategy to enhance vaccine responses (Linterman and Hill, 2016). In this clinical trial, only volunteers receiving the vaccine containing GLA-SE had an expansion of the ICOS^+^CD38^+^CXCR5^+^PD-1^+^ cTfh cell population 7 d after the third vaccination (Fig. 3, A–C), and the increase in cTfh cell frequency correlated positively with an increase in anti-P27A IgG at peak response (Fig. 3 D). This demonstrates that altering the vaccine adjuvant is a rational approach to enhance the magnitude of the cTfh cell response in humans. These data show that GLA-SE can provoke a robust boost of humoral immunity even in the context of high preexisting antibody titers induced by the previous two P27A inoculations. This finding is critical in cases where preexisting natural antigen exposure may have a negative impact on vaccine responses, such as in malaria-endemic regions like Tanzania.

### Public TCRβ clonotypes identified in cTfh cells from GLA-SE–inoculated individuals

**Figure 4.**
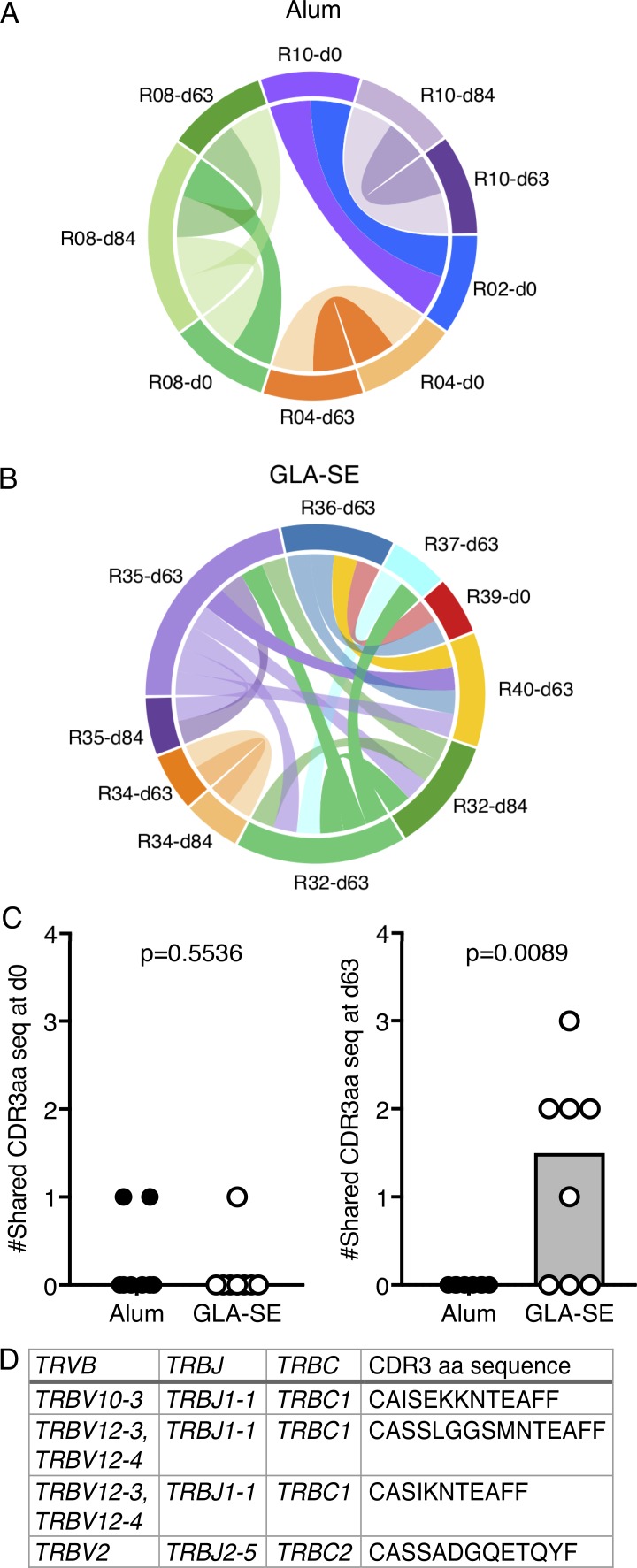
**A GLA-SE adjuvanted vaccine elicits common TCRβ clonotype usage. (A and B)** Circos plots of shared TCRβ CDR3 amino acid sequences in CD38^+^ICOS^+^CXCR5^+^PD-1^+^ cTfh cells from donors who received the P27A vaccine in (A) Alum or (B) GLA-SE. Alum group, *n* = 7; GLA-SE group, *n* = 8. Each line represents a TCRβ CDR3 amino acid sequence shared with another sample, and a different color was used for each participant. **(C)** Quantification of the number of shared CD38^+^ICOS^+^CXCR5^+^PD-1^+^ cTfh TCRβ CDR3 amino acid sequences per donor either before vaccination (P = 0.5536; left, sharing between day 0 samples) or 7 d after the third vaccination (P = 0.0089; right, sharing between day 63 samples). Alum group, *n* = 7; GLA-SE group, *n* = 8. **(D)** TCRβ gene usage and CDR3 amino acid sequence of the TCRβ CDR3 clonotypes shared between GLA-SE–vaccinated individuals at day 63. P values were calculated using a two-tailed unpaired Student’s *t* test. Each symbol represents one individual; those who received Alum are shown in black, and those who received GLA-SE are in white. Data are from one clinical trial.

As GLA-SE quantitatively altered the cTfh cell response in humans, we wanted to determine if it also has a different qualitative effect compared with Alum on this cell population. Previous studies have shown that CXCR5^+^CD45RA^−^CD4^+^ cTfh cells have B cell helper capacity in vitro (Simpson et al., 2010; Chevalier et al., 2011; Morita et al., 2011; Bentebibel et al., 2013; He et al., 2013; Locci et al., 2013). In this study, <500 ICOS^+^CD38^+^ cTfh cells were isolated per person, meaning coculture studies to address the functional capacity ex vivo could not be pursued, but low-cell-number RNA sequencing was still achievable. As established in our UK influenza vaccination study (Fig. 1), we performed total RNA-sequencing on 150–200 ICOS^+^CD38^+^ cTfh cells per person before vaccination (day 0), and 7 (day 63) and 28 (day 84) d after the third vaccination. There was no significant alteration in the cTfh cell transcriptome between adjuvant groups (Alum, *n* = 7; GLA-SE, *n* = 8) 7 d after the third vaccination (Fig. 3 E) or at the other time points analyzed (data not shown). This suggests that while the GLA-SE–formulated peptide increases the magnitude of the cTfh cell response, it is not likely to alter cTfh cell function when compared with Alum. Consistent with this, there was no difference in the mRNA expression of the key cTfh effector molecules *CD40LG* or *IL21* between the two volunteer groups (Fig. 3, F and G). In contrast to what was observed in the UK unadjuvanted influenza vaccination cohort, there was no significant change in the *CXCR3* or *CCR6* transcripts (Fig. 3, H and I), indicating that the Th1/2/17 phenotype of these cTfh cells is not skewed by either adjuvant. This observation is consistent with the similar distribution of P27A-specific IgG subclasses found after vaccination (Fig. 2 F). To determine whether adjuvant choice had an effect on the clonality of the cTfh response, we examined TCRβ sequences in our RNA-sequencing dataset of samples from days 0, 63, and 84. There was no evidence of clonal expansion in either adjuvant group (Fig. S3), but common clones were identified between samples. Within the Alum group, we identified 10 TCRβ clonotype sequences that were shared between samples (five distinct clones, each present in two separate samples); however, with one exception, these were only shared within individuals at different time points (Fig. 4 A). In the GLA-SE–vaccinated group, of the nine distinct TCRβ clones that were common between two different samples, six were public TCRβ clonotypes, shared between five different individuals (Fig. 4 B). Strikingly, these shared TCRβ clonotypes were not commonly detected in samples from baseline and day 84 but were identified at the peak of the cTfh response (day 63) in five of eight GLA-SE–vaccinated individuals (Fig. 4, C and D). These data indicate that GLA-SE may either promote the recruitment or selective expansion of T cells expressing shared TCRβ clonotypes into the cTfh cell compartment.

### GLA-SE–formulated vaccines stimulate the extrafollicular ASC response

**Figure 5.**
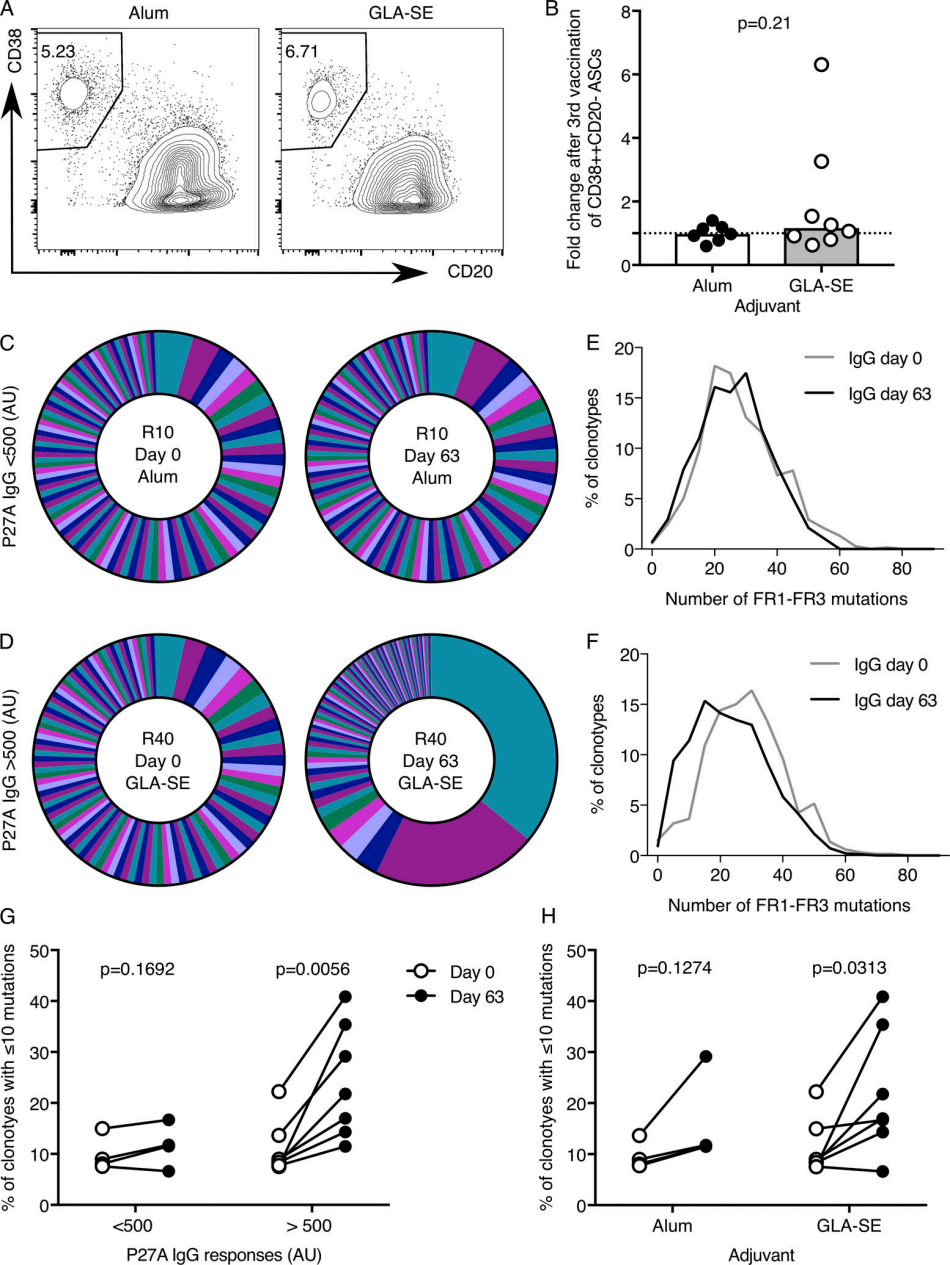
**V(D)J sequencing shows low BCR mutation frequency in individuals with high titer antibody responses. (A and B)** Flow cytometric contour plots (A) and quantitation (B) of peripheral blood CD38^+^CD20^−^ cells of total CD19^+^ cells 7 d after the third vaccination (day 63). Alum group, *n* = 7; GLA-SE group, *n* = 8. P = 0.21. In B, the height of the bar represents the median. P values were calculated using a Mann-Whitney *U* test, and each symbol represents one individual: those who received Alum are shown in black, and those who received GLA-SE are in white. **(C and D)** Pie charts of the proportions of the 100 most abundant IgHG clonotypes in CD38^+^CD20^−^CD19^+^ ASCs from C, a representative individual whose anti-P27A IgG does not increase >500 AU after the third vaccination (day 84–day 0), and D, a representative individual who has a high anti-P27A titer after the third vaccination (day 84 to day 0). Each segment of the pie chart represents a unique BCR clonotype. **(E and F)** Line graphs of the number of mutations in the V region of each clonotype (FR1–FR3, excluding CDR3, binned into five mutation bins) for the individuals shown in A and B, respectively, at the indicated time points relative to vaccination. **(G and H)** The percentage of IgHG clonotypes with ≤10 mutations in low (*n* = 4; P = 0.1692) and high antibody responders (*n* = 7; P = 0.0056; G) and in the different adjuvant groups, Alum group *n* = 4, P = 0.1274; GLA-SE group *n* = 7, P = 0.0313 (H). In G and H, each individual is connected with a line between their day 0 and day 63 samples. The P values were from a paired Student’s *t* test. Individual participants’ clonotype data for all other samples that passed sequencing quality control are included as Fig. S4. Data are from one clinical trial.

Because GLA-SE promoted the expansion of cTfh cells, we wanted to determine whether this adjuvant also has an impact on the B cell response after vaccination. To this end, we assessed the number and the repertoire of peripheral blood ASCs. Despite the increase in P27A antibody titers (Fig. 2, B and C), there was not an increase in the frequency of circulating CD38^+^CD20^−^CD19^+^ ASC 7 d after the third vaccination (day 63) in either group relative to baseline (Fig. 5, A and B). This may be because it is difficult to detect numerical changes in blood ASCs in Tanzanian individuals due to higher baseline frequencies than in age-matched samples from our UK cohort (Fig. S4 A). Alternatively, this may be because 7 d after the third vaccination (day 63) is not the peak of the circulating ASC response in this vaccine trial. An alternative strategy to understand the detailed nature of B cell responses in humans after vaccination is to assess the clonality and mutation rate in ASCs. To investigate the characteristics of vaccine-induced ASCs, we performed V(D)J RNA sequencing of immunoglobulin heavy chain IgG (IgHG) repertoires in blood ASCs from all individuals 7 d after the third vaccination (day 63). The analysis revealed that individuals with high titer antibody responses (defined as anti-P27A IgG >500 arbitrary units [AU] at day 84) had oligoclonal expansion of ASC clones 7 d after the third vaccination (Fig. 5, C and D; and Fig. S4 B). Strikingly, these individuals showed an enrichment of IgHG clonotypes with fewer somatic mutations in their IgG heavy chain variable region (FR1–FR3 regions), compared with the blood sample obtained before vaccination (Fig. 5, E–H). This indicates that high-titer responders have more circulating ASCs derived from the extrafollicular antibody response than low responders. This decreased frequency of mutations occurred more frequently in GLA-SE–vaccinated individuals than in those that received the Alum-formulated P27A peptide (Fig. 5 H), suggesting that GLA-SE stimulates extrafollicular ASC responses more efficiently than Alum. This observation is consistent with a murine study showing that GLA-SE stimulates a greater number of antigen-specific plasmablasts in the draining LN after immunization than an Alum-formulated vaccination (Desbien et al., 2016). It is important to note that this occurs in parallel with an enhancement of the GC response, demonstrating that both responses can be simultaneously enhanced by GLA-SE in mice and that this adjuvant does not skew the B cell response in favor of one pathway over the other (Desbien et al., 2016; Olafsdottir et al., 2016).

### Transcriptional analysis identifies circulating counterparts of GC-Tfh cells

**Figure 6.**
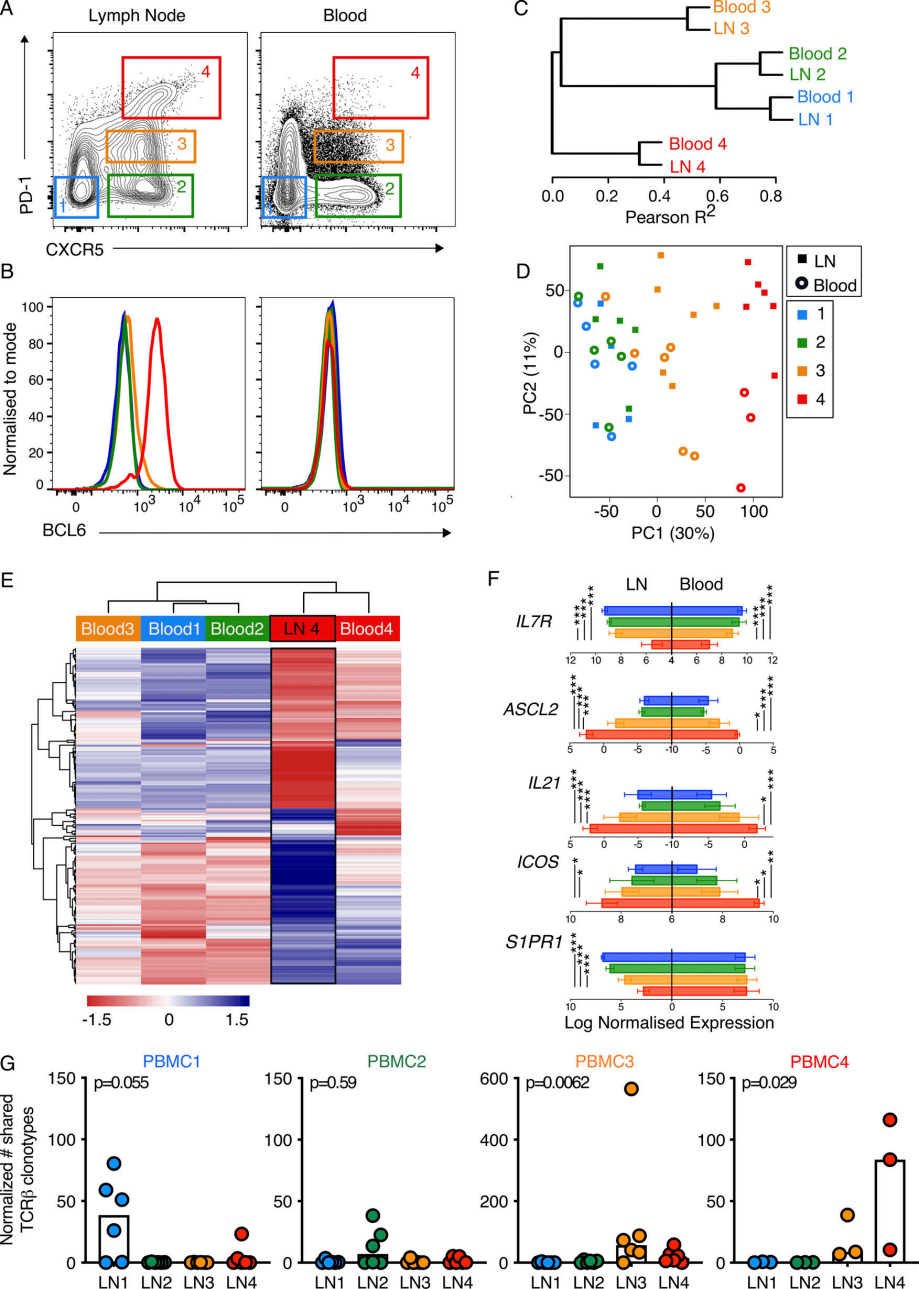
**Transcriptional analysis identifies circulating counterparts of GC-Tfh cells. (A and B)** Flow cytometric plots showing the gating strategy of the four populations of CD4^+^CD3^+^CD45RA^−^ cells sorted from iliac LNs (left) and peripheral blood (right) for RNA-sequencing and the expression of BCL6 protein in these populations, with representative plots shown; *n* = 6 individuals. **(C and D)** Hierarchical clustering (C) and PC analysis (D) of the most highly variable transcripts (standard deviation, >0.7) from RNA-sequencing data from four populations sorted in A from blood and LN samples. Three peripheral blood samples from the blood population no. 4 were excluded from the analysis as they did not pass quality control. In D, each symbol represents one individual. **(E)** Supervised analysis of the RNA-sequencing data from the four populations isolated from the blood using a gene signature for the LN GC-Tfh cells (LN 4); *n* = 254 genes. **(F)** Expression of *IL7R*, *ASCL2*, *IL21*, *ICOS*, and *S1PR2* mRNA from RNA-sequencing data of the populations sorted in A; cell types are indicated by the colors shown in A. RNA-sequencing data were standardized relative to a library size of 1 million reads, and then read counts were log2 transformed; a value of −5 represents the limit of detection. P values were determined using ANOVA and the Tukey multiple-comparison test (*, P < 0.05; **, P < 0.01; ***, P < 0.001). **(G)** Quantification of the number of TCRβ CDR3 amino acid sequence clonotypes found in each blood cell population that are shared with the different LN populations (for each cell type *n* = 6 donors, except blood population no. 4 where *n* = 3; shared numbers of clonotypes were converted to normalized pseudocounts as described in Statistical analyses and Materials and methods). PBMC1, P = 0.055; PBMC2, P = 0.59; PBMC3, P = 0.0062; PBMC4, P = 0.029. The P values were determined using Kruskal-Willis tests. Data are from six individuals in one experiment.

In both mice and humans, Tfh cells play essential roles within the GC (GC-Tfh) and outside the B cell follicle supporting the extrafollicular antibody response (McHeyzer-Williams et al., 2009; Bentebibel et al., 2011; Lee et al., 2011). Our results indicate that GLA-SE is able to promote both cTfh cell and extrafollicular antibody responses, prompting the hypothesis that using ICOS^+^CD38^+^CXCR5^+^PD-1^+^ cTfh cells as a biomarker of Tfh cells, as in our study, does not discriminate between GC-Tfh and extrafollicular Tfh cells, which would both be expected to form after vaccination to support their respective B cell responses. Recent studies suggest that cTfh cells with the highest expression of PD-1 represent a highly activated cTfh cell population (Schmitt et al., 2014; Heit et al., 2017), and their similar phenotype to LN GC-Tfh suggests that CXCR5^+^PD-1^+++^ cTfh cells may represent a distinct biomarker of GC-Tfh cells. To test this hypothesis, four different CD4^+^CD45RA^−^ T cell subsets were sorted based on their expression of CXCR5 and PD-1 (gating shown in Fig. 6 A), including bona fide LN CXCR5^+^PD-1^+++^Bcl-6^+^ Tfh cells (Fig. 6 B), from paired human blood and iliac LN samples from six individuals, and their transcriptomes were sequenced as described above. Hierarchical clustering of the most variable genes shows that CD4^+^CD45RA^−^ T cell subsets from blood clusters with their corresponding subset from LNs expressing the same cell surface phenotype (Fig. 6 C). Principal component (PC) analysis shows that majority of variation in the data is driven by the cell phenotype (PC1, 30%) while the second contributor to variation (PC2, 11%) is tissue origin (Fig. 6 D). Together, this strongly suggests that the CXCR5^+^PD-1^+++^ cTfh population most closely resembles the LN resident CXCR5^+^PD-1^+++^Bcl6^+^ GC-Tfh cell type. To further determine to what extent the transcriptome of the cTfh cells overlaps with that of the GC-Tfh population (LN 4, Fig. 6 A), we first generated a list of 254 genes that were differentially expressed in GC-Tfh (LN 4) compared with the three other LN populations (LN 1, 2, 3), then used this gene signature to compare with the four different blood CD4^+^CD45RA^−^ populations (Fig. 6 E). The CXCR5^+^PD-1^+++^ cTfh population clustered most closely with the LN GC-Tfh cell population, with a number of gene modules showing the same expression pattern (Fig. 6 E); for example, the canonical Tfh genes *ASCL2*, *IL21*, and *ICOS* show the same expression patterns in both LN and circulating CXCR5^+^PD-1^+++^ cells (Fig. 6 F). Notably, *IL7R* expression is low in both LN and circulating CXCR5^+^PD-1^+++^ cells (Fig. 6 F), consistent with a GC-Tfh phenotype (Bentebibel et al., 2011). However, there are differences between the LN and circulating populations, as exemplified by low expression of *S1PR1* on LN-GC-Tfh cells but high expression on CXCR5^+^PD-1^+++^ cTfh cells (Fig. 6 F), consistent with its role as a blood-localizing receptor on T cells. Analysis of the TCRβ CDR3 sequences from the paired blood and LN cell populations revealed that a high proportion of CDR3 sequences were shared between CXCR5^+^PD-1^+++^ cTfh and GC-Tfh cells (Fig. 6 G), consistent with a previous study that sequenced the TCR of PD-1^+^ICOS^+^ cTfh cells (Heit et al., 2017). Combined, our data demonstrate that CXCR5^+^PD-1^+++^ cTfh cells are clonally and transcriptionally most closely related to GC-Tfh in secondary lymphoid tissues; this shared ontology and antigen specificity mean this cTfh population can be used as a surrogate for GC-Tfh cells.

### The expansion of CXCR5^+^PD-1^+++^ cTfh cells is enhanced in GLA-SE–vaccinated participants

**Figure 7.**
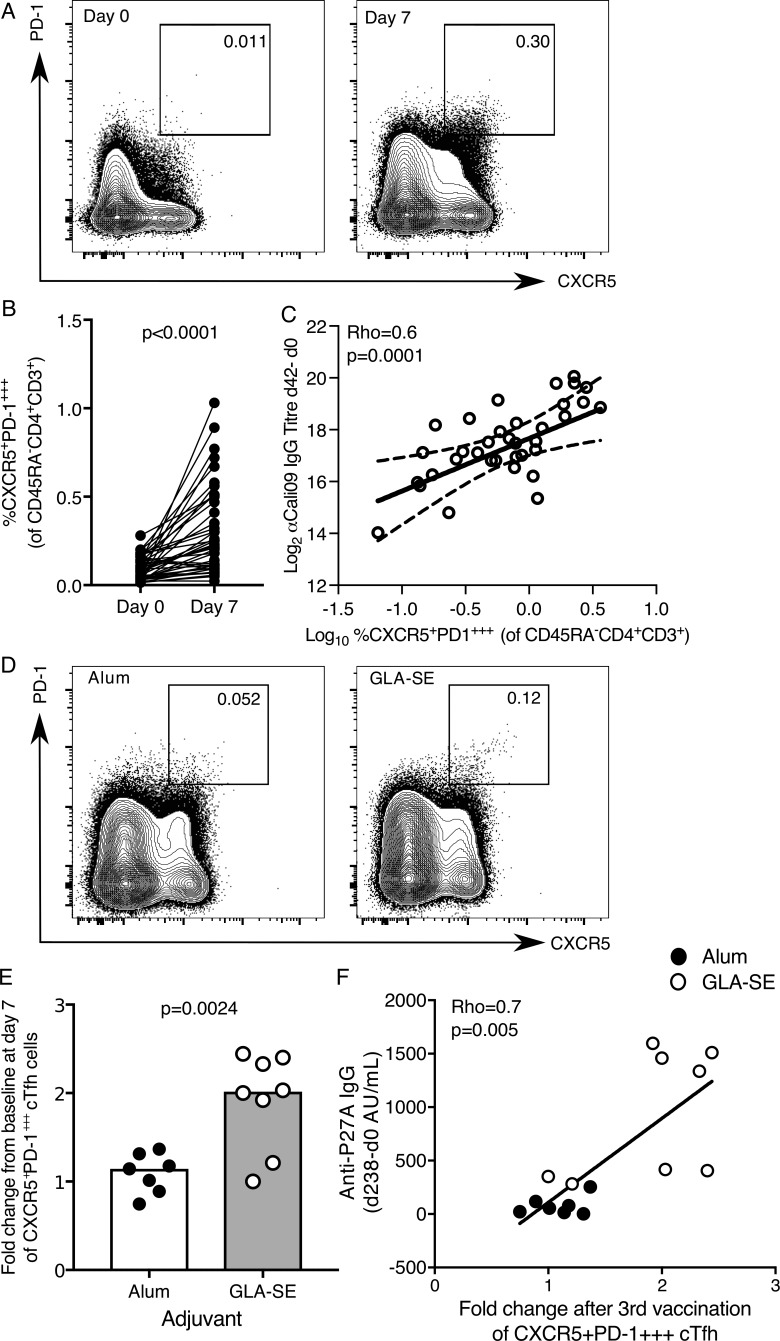
**The expansion of CXCR5^+^PD-1^+++^ cTfh cells is enhanced by a GLA-SE adjuvanted vaccine. (A and B)** Flow cytometric contour plots (A) and quantification (B) of the frequency of CXCR5^+^PD-1^+++^ cells among CD45RA^−^CD4^+^CD3^+^ cells in the peripheral blood of healthy UK donors at days 0 and 7 relative to seasonal influenza vaccination; *n* = 41; in B, each symbol represents a volunteer, and an individual donor is connected by a line at the two time points; *n* = 41. P < 0.0001. The P value was calculated using Wilcoxon signed-rank test. **(C)** Correlation of the frequency of CXCR5^+^PD-1^+++^ cTfh cells 42 d after vaccination with the change in antibody titer of anti-Cal09 IgG (an influenza A HA); Rho = 0.6; P = 0.0001 using Spearman’s correlation (Rho = correlation coefficient). **(D)** Flow cytometric contour plots of CXCR5^+^PD-1^+++^ of total CD4^+^CD3^+^CD45RA^−^ cells 7 d after the third P27A vaccination. Alum group, *n* = 7; GLA-SE group, *n* = 8. **(E)** Fold change of CXCR5^+^PD-1^+++^ cTfh cells 7 d after the third P27A vaccination (frequency of cTfh cells at day 63/baseline). Alum group, *n* = 7; GLA-SE group, *n* = 8. P = 0.0024. The P value is from an unpaired Student’s *t* test. **(F)** Correlation of the increase in anti-P27A IgG antibody titer 238 d after the first vaccination (relative to baseline) against the fold change in CXCR5^+^PD-1^+++^ cTfh cells 7 d after the third vaccination (day 63); *n* = 15. Rho = 0.7; P = 0.005 using Spearman’s correlation. Each symbol represents one individual; those who received Alum are shown in black, and those who received GLA-SE are in white. Data are from one clinical trial.

The direct comparison of paired blood and LN samples indicated that CXCR5^+^PD-1^+++^ cTfh cells may be a circulating biomarker of GC-Tfh cells. Next, we tested whether CXCR5^+^PD-1^+++^ cTfh expansion correlated more with vaccination-induced long-term IgG responses measured after influenza and P27A vaccination, as long-term antibody responses are GC derived. CXCR5^+^PD-1^+++^ cTfh cells expanded after seasonal influenza vaccination in our UK cohort, confirming their responsiveness to immunization (Fig. 7, A and B; Carr et al., 2016; Heit et al., 2017) and the presence of HA-specific T cells within this population was confirmed (Fig. S2, D and E). This expansion also correlates with the increase in antibody titer 42 d after vaccination (Fig. 7 C). To determine the effect that GLA-SE has on this population, we reanalyzed our flow cytometry data from the P27A vaccine trial with this alternate gating strategy. There was a significant expansion of CXCR5^+^PD-1^+++^ cTfh cells in the GLA-SE group but not in those volunteers who received the Alum-formulated peptide (Fig. 7 E). Importantly, the increase in CXCR5^+^PD-1^+++^ cTfh cells at day 63 correlated with long-term antibody titers assessed on day 238 (Fig. 7 F), suggesting that this population may promote the acquisition of long-term humoral immunity, a property consistent with stimulating a GC response. These data, together with murine research demonstrating GLA-SE promotes GC B cell number in the draining LN (Desbien et al., 2016), suggest that GLA-SE is a rational choice of adjuvant for enhancing the GC response and long-lasting humoral immunity in humans.

## Discussion

Adjuvants are currently the most tractable way of altering how the human immune system responds to vaccination. The majority of licensed vaccines provide protection against subsequent infection by generating long-lived antibody responses. Therefore, adjuvants that enhance the magnitude of the GC response are a logical approach to enhancing humoral immunity. The size and quality of the GC response depend on the quantity of Tfh cells that are induced by vaccination. While animal studies clearly show that different adjuvants can enhance the Tfh cell response (Aloulou et al., 2016; Desbien et al., 2016), these findings need to be translated into human studies. Here, we show that a next-generation adjuvant can be used to enhance the magnitude of the Tfh cell response in humans. The GLA-SE adjuvant increases both the ICOS^+^CD38^+^CXCR5^+^PD-1^+^ total cTfh cell population and the GC-Tfh–like CXCR5^+^PD-1^+++^ cell population after P27A vaccination, indicating that this adjuvant enhances the GC response. The analysis of the ASCs by V(D)J sequencing showed that GLA-SE also enhances the extrafollicular response. The data presented here are consistent with a mouse immunization study showing that antigen formulated with GLA-SE enhances Tfh cell numbers, the GC reaction, and plasmablasts in secondary lymphoid tissues after immunization (Desbien et al., 2016). Together, this demonstrates that GLA-SE enhances the magnitude of the humoral immune response rather than skewing it toward either the GC reaction or the extrafollicular antibody response. The stimulation of both these ASC sources in parallel maximizes the production of vaccine-specific antibodies. This demonstrates that GLA-SE can be used to make vaccines that potentiate Tfh cell and antibody responses in humans.

Rapid antibody production after vaccination is supported by the extrafollicular ASC response. During a primary response, extrafollicular ASCs usually do not exhibit strong SHM in their antibody variable (V) gene segments because they do not derive from GCs. Importantly, our study was done on malaria-preexposed volunteers who received three P27A vaccinations at monthly intervals. Therefore, ASCs measured after the third vaccination could have differentiated from memory B cells derived either from a GC reaction after the first two P27A immunizations or from a natural malaria infection. These memory B cell–derived ASCs would be expected to have fewer somatic mutations than GC-derived ASCs because memory B cells emerge from the GC before long-lived plasma cells (Weisel et al., 2016). Consistent with this, individuals with high titer antibody responses 7 d after the third vaccination had mutations present in the FR1–FR3 regions of the antibody V gene segments; however, the frequency of mutations was lower than ASCs at baseline. This indicates these ASCs were likely of extrafollicular origin and suggests that the precursors of these ASCs were memory B cells that underwent low-level SHM during a GC response to one of the previous vaccinations in the vaccine trial. This FR1–FR3 region low-mutation signature was more prevalent in individuals receiving the GLA-SE–formulated vaccine and is consistent with the number of mutations reported in memory B cells (DeWitt et al., 2016) and memory B cell–derived ASCs after seasonal influenza vaccination in humans (Ellebedy et al., 2016). Studies in mice have shown that GLA-SE augments the formation of early ASCs in the draining LN (Desbien et al., 2016; Olafsdottir et al., 2016), and our findings indicate that in humans GLA-SE can also enhance the recruitment of memory B cells into an extrafollicular ASC response.

The generation of humoral immunity after vaccination occurs in secondary lymphoid tissues, which has hindered our ability to study detailed cellular vaccine responses in humans. Since the first descriptions of the expansion of a population of cTfh cells in the blood in patients with autoimmunity (Simpson et al., 2010) and subsequently after vaccination (Bentebibel et al., 2013; He et al., 2013), there has been growing interest in using cTfh cells as a biomarker of the GC response in humans (Linterman and Hill, 2016). A single unifying definition of what a Tfh cell is or a description of how they should be identified has yet to be precisely demarcated. It has been proposed that Tfh cells are defined by their capacity to provide help to B cells, and those that do so in the context of a GC reaction are described as GC-Tfh cells, thereby subsetting the total Tfh cell population by the site at which it functions (McHeyzer-Williams et al., 2009; Bentebibel et al., 2011; Lee et al., 2011). Here, we describe that a similar compartmentalization can be applied to the circulating counterparts of Tfh cells. The ICOS^+^CD38^+^CXCR5^+^PD-1^+^ cTfh cells represent a larger group of B cell helper cells; within this cTfh population, those cells with the highest expression of PD-1 are the most similar to the GC-Tfh cells found in human LNs, consistent with previous work (Heit et al., 2017). Analysis of the transcriptome of cTfh cells has the potential to yield information about how vaccination changes the phenotype of Tfh cells. We observed changes in the transcriptome of these cells 7 d after seasonal subunit influenza vaccination in UK adults. These included alterations in the expression of *CXCR3* and *CCR6*, chemokine receptors known to be affected by this vaccination (Bentebibel et al., 2013), demonstrating that RNA sequencing is a viable approach to detect phenotypic changes in cTfh cells after vaccination. However, our study did not detect differences in the transcriptomes of ICOS^+^CD38^+^CXCR5^+^PD-1^+^ cTfh cells 7 d after the third inoculation with a synthetic peptide formulated in Alum and GLA-SE, suggesting that the main difference in cTfh cells driven by these adjuvants is quantitative rather than qualitative. This quantitative difference may arise from the distinct mechanisms of action of the two adjuvants; while GLA-SE is thought to exert its effect on subcapsular sinus macrophages via IL-18 and TLR4 (Desbien et al., 2016), Alum does not act through TLRs but instead may act via the inflammasome (Eisenbarth et al., 2008). The stable emulsion (SE) in GLA-SE has adjuvant properties on its own, such as is seen for emulsion adjuvants like MF59. It is clear from preclinical studies that the combination of GLA with SE creates a more potent stimulator of both humoral immunity and the Tfh cell response than either component individually (Desbien et al., 2016; Knudsen et al., 2016). Therefore, it is likely that this combination adjuvant is responsible for supporting the enhanced cellular immune response described in this study. Our results indicate that, despite different mechanisms of innate immune cell activation by these two adjuvants, they do not affect innate immune cells in a way that results in a qualitatively different Tfh cell response.

Our transcriptomic analysis revealed that GLA-SE also promotes the emergence of common TCRβ clonotypes in cTfh cells that were shared between five of eight individuals. The emergence of so-called public clonotypes is informative for vaccine design as it indicates that there may be a common, immune-dominant epitope that could be used to specifically enhance T cell responses in human populations. The recent discoveries of public antibody clonotypes have revolutionized whole-parasite malaria vaccine strategies (Imkeller et al., 2018; Tan et al., 2018), and here, we extend this to the identification of public cTfh cell TCRβ clonotypes induced after vaccination. The identification of these common TCRβ clonotypes in cTfh cells from participants who received the GLA-SE–formulated vaccine could be a consequence of the greater magnitude of the response, indicating that more T cell clones are recruited into the cTfh cell response. The knowledge of key antigenic peptides to which multiple individuals respond, combined with adjuvants that allow a better recruitment of Tfh cells into the GC, outlines a rational way to enhance vaccine responses at the population level.

To date, there have been multiple subtypes of cTfh cells identified that have different activation states and functional capacities (Morita et al., 2011; Bentebibel et al., 2013; Locci et al., 2013; Schmitt et al., 2014). Here, we aimed to identify the population of cTfh cells that are most similar to GC-Tfh cells in human LNs. While GC-Tfh cells are likely to be the most informative cell type in vaccination trials in which the generation of long-lived somatically mutated GC-derived ASCs is desirable, obtaining GC-Tfh cells is not possible in large vaccination studies. We show here that cTfh can provide surrogacy for GC-Tfh cells, including insights into the transcriptome of Tfh cells. CXCR5^+^PD-1^+++^ cTfh cells have a similar, although not identical, RNA profile to GC-Tfh cells found in LNs. These two cell types also shared TCRβ clonotypes, which indicates they may be clones derived from the same cell. This clonal relationship is consistent with a previous study (Heit et al., 2017), which together with the data presented here suggests that cTfh cells with high PD-1 expression are closely clonally and transcriptionally related to GC-Tfh cells. This study supports the relevance of studying the CXCR5^+^PD-1^+++^ cTfh population in this and future studies of how vaccines shape human GC-Tfh cell biology. Our finding that GLA-SE can enhance the GC-Tfh-like cTfh population and long-lived antibody response in the P27A vaccine trial demonstrates that the adjuvant should be a key consideration in vaccine design to maximize the generation of protective T cell–dependent humoral immunity in humans.

## Materials and methods

### Study design

The main research objective of this study was to characterize the cTfh cell and ASC responses to vaccination in humans at the cellular and molecular level. To that end, two related studies were performed: (1) peripheral blood was tested from 41 healthy UK adults (18–98 yr of age) who were vaccinated with the trivalent influenza vaccine (northern hemisphere winter 2016–2017) and (2) 16 healthy HIV-negative Tanzanian male adults (18–45 yr old) with minimal malaria exposure (urban Dar-es-Salaam), who were vaccinated with 50 µg P27A peptide formulated in either Alhydrogel (*n* = 8) or 5 µg GLA-SE (*n* = 8). Serum was available for all participants for analysis, and peripheral blood mononuclear cells (PBMCs) were available from seven in the Alhydrogel-vaccinated group and eight in the GLA-SE–vaccinated group (Steiner-Monard et al., 2019). Circulating Tfh cells and ASCs were identified by flow cytometry in samples before and after vaccination and were flow-sorted for mRNA or B cell receptor (BCR) repertoire sequencing, respectively. Researchers remained blinded to the adjuvant group throughout sample processing and data acquisition. Tonsil samples were collected from adults undergoing routine tonsillectomy at Cambridge University Hospitals NHS Foundation Trust. Paired blood and LN samples were taken from patients recruited from the renal transplant live donor program at Cambridge University Hospitals NHS Foundation Trust and who provided informed consent. All patients were either receiving or within 6 mo of requiring renal replacement therapy. Patients taking immunosuppressive medication before the transplant were excluded. 50 ml of blood was taken at the time of transplant, before knife to skin. Lymphoid tissue was removed as part of the routine operative procedure as previously described (Wallin et al., 2014). All human blood and tissue were collected in accordance with the latest revision of the Declaration of Helsinki and the Guidelines for Good Clinical Practice. The seasonal UK influenza vaccination cohort was collected with UK local research ethics committee (REC) approval (REC reference 14/SC/1077), using the facilities of the Cambridge Bioresource (REC reference 04/Q0108/44). The P27A vaccine phase Ib trial (ClinicalTrials.gov Identifier: NCT01949909, Pan African Clinical Trial Registry identifier: PACTR201310000683408) was conducted with approval from the Tanzanian Food and Drug Administration (TFDA; Dar-es-Salaam, TFDA13/CTR/004/03), National Institute for Medical Research (NIMR; Dar-es-Salaam, NIMR/HQ/R8a/Vol.IX/1742), Swiss Agency for Therapeutic Products (Swissmedic, Bern, Switzerland, reference no. 2013DR1165), and ethical review boards at Ifakara Health Institute and the University of Lausanne. Use of P27A trial samples in the UK was approved by the UK Health Research Authority (REC reference 17/EE/0063) and Babraham Institute Human Ethics Committee. Tonsil tissues and paired blood and LN samples were collected from UK adults undergoing surgery for their own medical care, under ethical approval from UK Health Research Authority (REC references 16/LO/0453 and 11/EE/0355, respectively) at Cambridge University Hospitals and processed at the Babraham Institute. Written informed consent was received from all volunteers.

### Cell isolation of PBMC, LN, and tonsillar lymphocytes

Blood samples were collected into EDTA-coated tubes on the day of vaccination (before administration of the vaccine) and at the indicated time points. PBMCs were isolated using 15 ml Histopaque-1077 (Sigma-Aldrich) then frozen in fetal bovine serum supplemented with 10% dimethyl sulfoxide (Sigma-Aldrich) overnight in a methanol bath at −80°C, then kept in liquid nitrogen before analysis by flow cytometry. To isolate a single cell suspension from secondary lymphoid tissues, tonsil and LN samples were finely minced and then pressed through a 70-µm cell strainer (Becton Dickinson), cells were washed twice in 50 ml sterile PBS, then frozen as above.

### Flow cytometry and sequencing of cTfh cells, tissue Tfh cells, and ASCs

Cryopreserved mononuclear cells were thawed and rested for 1 h at 37°C. Cell types were pre-enriched using MagniSort CD19 Positive Selection followed by CD4 memory T cell enrichment (eBioscience). Fc receptors on all cells were blocked using human IgG, followed by staining with panels outlined in Table 1 and separation on BD Biosciences Aria Fusion or Influx cell sorters. A dump channel consisting of viability dye and antibodies to CD14, CD16, and either CD19 or CD3 was used to exclude unwanted cell types from cTfh cell and ASC sorts, respectively. HA-specific CD4^+^ T cells were identified using class II tetramers with methods and reagents that have been previously reported (Yang et al., 2013). Gating strategies for ICOS^+^CD38^+^PD-1^+^CXCR5^+^ cTfh cells and ASCs are included as Fig. S5. Tonsil and LN cells were stained with the same flow cytometry panel as cTfh cells, and ∼4 million cells were costained for intracellular Bcl6 (Clone K112-91) after permeabilization with the transcription factor fixation/permeabilization kit (Life Technologies). mRNA was isolated from sorted cTfh cells (CD4^+^CD45RA^−^CXCR5^+^PD1^+^ICOS^+^CD38^+^ cells) using the SMART-Seq V4 Ultra-Low Input RNA kit (Takara Bio) and sorting 200 cells directly into lysis buffer. mRNA from paired blood and LN CD4^+^CD45RA^−^ T cell populations was isolated from 52–1,000 cells sorted into lysis buffer. cDNA libraries were subsequently generated using the Nextera XT DNA Library Prep Kit (Illumina), followed by sequencing on the Illumina HiSeq 2000 with ∼50 million 100-bp single-end reads per sample. ASCs (CD19^+^IgD^−^CD27^+^CD71^+^CD20^−^CD38^+^) were sorted into RNAlater (500–10,000 cells) and RNA isolated using the RNeasy Micro kit (Qiagen). In all samples for which sufficient RNA was extracted, immunoglobulin heavy (IgG, IgM) chains were amplified using 5′RACE with unique molecular identifiers as previously reported (Turchaninova et al., 2016; Davydov et al., 2018) using Q5 High-Fidelity DNA Polymerase (New England BioLabs) and sequencing on the Illumina MiSeq (340 × 280-bp paired-end).

**Table 1.** Antibodies used for flow cytometry

**Marker**	**Clone**	**Fluorochrome**	**Company**	**Catalog no.**
Viability dye	n/a	eFluor780	eBioscience	65-0865-14
CD14	61D3	APC-eF780	eBioscience	47-0149-42
CD16	eBioCB16	APC-eF780	eBioscience	47-0168-42
CD3	UCHT1	APC-eF780	eBioscience	47-0038-42
CD19	HIB19	BB515	BD Biosciences	564456
CD20	2H7	PECY7	BioLegend	302312
CD71	CY1G4	PE	BioLegend	334106
IgD	IA6-2	BV421	BD Biosciences	563813
CD27	M-T271	BV510	BioLegend	356420
CD38	HIT2	APC	eBioscience	17-0389-42
CD19	HIB19	APC-eF780	eBioscience	47-0199-42
ICOS	ISA-3	APC	eBioscience	17-9948-42
CD3	UCHT1	BUV 395	BD Biosciences	563546
CD45RA	HI100	BUV737	BD Biosciences	564442
CXCR5	RF8B2	BB515	BD Biosciences	564624
CD4	RPA-T4	PercpCy5.5	BD Biosciences	560650
PD1	eBioJ105	PECy7	eBioscience	25-2799-42
CD38	HIT2	BV421	BioLegend	303525
CD127	A019D5	BV650	BioLegend	351325
BCL6	K112-91	PE	BD Biosciences	561522
CXCR3	1C6/CXCR3	BV421	BD Biosciences	562558
CCR6	11A9	BV786	BD Biosciences	563704

n/a, not applicable.

### Serology

IgG to influenza HA proteins were measured before and after vaccination by Luminex using magnetic beads coated with full-length recombinant HA proteins from A/California/07/2009 (Cali09) and B/Brisbane/60/2008 (Bris08) as previously reported (Wang et al., 2015). IgG to P27A was measured by ELISA as previously reported (Steiner-Monard et al., 2019). Titers are represented as arbitrary units per milliliter, and where indicated, preexisting IgG titers were subtracted to calculate vaccination-induced IgG responses.

### Differential gene expression analysis

Transcriptomic analyses of cTfh cells and LN Tfh cell populations were performed using the SeqMonk software package (Babraham Institute, https://www.bioinformatics.babraham.ac.uk/projects/seqmonk/) after alignment of reads to the reference human genome GRCh38 using HISAT2 (Kim et al., 2015). Reads were quantitated over exons, the library size was standardized to 1 million reads, and then read counts were log2 transformed. Differentially expressed genes were determined by DESeq2 using raw counts (adjusted P value cutoff, <0.05; Love et al., 2014). Hierarchical clustering and PC analysis were performed using variable genes (those with a standard deviation of >0.7 when comparing all the datasets). The LN GC-Tfh signature was generated using DESeq2 and applying a post hoc z-score threshold of 0.9 to select the genes most highly or least expressed in GC-Tfh cells relative to other LN populations.

### TCRβ clonotyping and V(D)J sequencing

TCRβ clonotypes were called from adaptor-trimmed RNA sequencing fastq files using MIXCR (version 2.1.9; Bolotin et al., 2015) run in RNA-Seq mode (mixcr align -p rna-seq -c TRB -s hsa -OallowPartialAlignments = true) with rescuing of partial alignments and set to collate clonotypes at the amino acid level rather than the nucleotide level and requiring more than five reads to identify a clonotype. Clonotype diversity and sharing were determined using vdjtools (version 1.0.3; Shugay et al., 2015). Processing and analysis of the V(D)J sequencing reads of ASCs were done using MIGEC and MiXCR as described in Turchaninova et al. (2016) but with some changes. Briefly, “migec Histogram” was used as described but with omitting the “--only-first-read” command. The recommended molecular identifier group (MIG)–size thresholding from this was used to select MIGs with sufficient over-sequencing to allow efficient error correction in the subsequent “migec Assemblebatch” process and “--only-first-read” was again omitted from “migec Assemblebatch.” Over-sequencing thresholds used ranged from 3–16 with the majority (27 of 30) being ≥4. The “kaligner” alignment algorithm was used in “mixcr align” to map reads to the “VTranscipt” feature as recommended for full-length V(D)J data. Vdjtools (version 1.1.7; Shugay et al., 2015) was used for summarizing and visualizing results. SHM was analyzed using IMGT HighV-QUEST (Brochet et al., 2008). Briefly, full-length (FR1–FR4) V region nucleotide sequences of clonotypes output by MiXCR were filtered to remove noncoding, incomplete (these were both always very-low-frequency clonotypes) and non-IgG clonotypes. The sequences of the remaining coding, complete IgG clonotypes were submitted to IMGT HighV-QUEST and SHM analyzed using the “V-REGION-nt-mutation-statistics” output file. CDR3 was omitted from this since SHM of this cannot be differentiated from changes that occurred during V(D)J recombination and SHM of FR4 is not analyzed by IMGT. Clonotype sizes and SHM frequencies were analyzed and visualized using Excel and Graphpad prism.

### Data availability

RNA-sequencing data of cTfh before and 7 d after influenza vaccination in the UK cohort presented in Fig. 1 are available from Array Express (accession no. E-MTAB-7955). RNA-sequencing data cTfh before and 7 and 28 d after P27A vaccination from the Clinical Trial presented in Figs. 3 and 4 are available from the Gene Expression Omnibus (GEO; accession no. GSE131088). V(D)J-sequencing data from ASCs before and 7 and 28 d after P27A vaccination from the clinical trial presented in Fig. 5 are available from GEO (accession no. GSE131090). RNA-sequencing data presented in Fig. 6 from different CD45RA^−^CD4^+^CD3^+^ T cell populations isolated from paired blood and LN samples are available from GEO (accession no. GSE131089).

### Statistical analyses

Shapiro-Wilk testing indicated that the cTfh cell frequencies follow a normal distribution in both the UK influenza vaccination cohort and Tanzanian P27A vaccination cohort. For the UK influenza vaccination studies, comparisons were made between days 0 and 7 for each individual with a paired Student’s *t* test. For the P27A study, comparisons between the Alum and GLA-SE groups were done using a two-tailed unpaired Student’s *t* test. ASC frequencies and serum IgG levels in the Tanzanian P27A vaccination cohort did not follow a normal distribution and were analyzed using a Mann-Whitney *U* test. Comparisons of the IgG titer from multiple time points in the Tanzanian P27A vaccination cohort were calculated on log-transformed data using a two-way ANOVA with Sidak’s multiple-comparisons test. The Kruskal-Willis test was used to compare TCRβ sharing in the paired blood and LN study. The number of cells sorted per population and the number of TCRβ clonotypes for the paired blood and LN study are shown in Table 2. Figure legends include the test and the number of observations.

**Table 2.** The numbers of individual samples that passed quality control, cells flow sorted for each population, and the corresponding number of TCRβ clonotypes identified at amino acid level

**Population**	**N**	**Number of sorted cells (median [IQR])**	**Number of TCRβ clonotypes (median [IQR])**
LN 1	6	1,000 (1,000–1,000)	332 (185–525)
LN 2	6	1,000 (1,000–1,000)	294 (264–386)
LN 3	6	1,000 (1,000–1,000)	455 (282–562)
LN 4	6	1,000 (1,000–1,000)	461 (403–558)
Blood 1	6	1,000 (1,000–1,000)	205 (145–276)
Blood 2	6	1,000 (1,000–1,000)	262 (195–298)
Blood 3	6	1,000 (1,000–1,000)	338 (229–415)
Blood 4	3	145 (134–172)	135 (117–145)

IQR, interquartile range.

For paired blood and LN analysis of TCRβ clonotyping, we used the following strategy to allow for the effect of the smaller cell numbers in blood “population 4” (ICOS^+^CD38^+^CXCR5^+^PD-1^+^) based upon a prior study (Shugay et al., 2013). This paper shows for 12 unrelated donor pairs that the number of shared clonotypes (*Y*) is directly proportional to the square of the number of clonotypes tested (*X*), such that *Y* = *aX^2^*, where *a* is a constant. This extends work from Robins et al. (2010), which used a similar power law to extrapolate TCRβ clonotyping to estimate the total size of the human naive CD8 pool. We, therefore, normalized our observed sharing frequencies between populations *i* and *j* (Y*_ij_*) as follows.

Normalized sharing count between populations *i* and *j*, *NormY_ij_* = *Y_ij_*/((*X_i_* + *X_j_*)/2)^2^ where *X_i_* and *X_j_* are the number of clonotypes identified in populations *i* and *j*, respectively. For some comparisons, *Y_ij_* was equal to 0. To allow normalization, we used pseudocounts (ψ*Y_ij_* = *Y_ij_* + 0.01):

$$Norm\psi Y_{ij}\;=\;\left({Y_{ij}}_{\;}+\;0.01\right)/{\left(\left({X_{i}}_{\;}+\;X_{j}\right)/2\right)}^{2}.$$

We calculated *NormψY* between all eight populations (8!/2!6! = 28 comparisons) for each individual.

### Online supplemental material

Fig. S1 shows the expression of CD38 and ICOS on tonsillar Tfh cells and the relationship of CD38^+^ICOS^+^CXCR5^+^PD-1^+^ Tfh cells with GC B cells. Fig. S2 shows HA-specific CD4^+^ T cells within the cTfh cell population 7 d after vaccination. Fig. S3 shows the proportion of the 40 most abundant TCRβ CDR3 clonotypes in ICOS^+^CD38^+^CXCR5^+^PD-1^+^ cTfh cells from each P27A study participant. Fig. S4 compares the frequency of ASCs before vaccination in both the UK and Tanzanian cohorts. Fig. S4 also shows the 100 most abundant BCR clonotypes in the CD38^2+^CD20^−^CD19^+^ ASCs of individual P27A study participants. Fig. S5 shows the gating strategy for fluorescence-activated cell sorting of cTfh cells and ASCs.

## Supplementary Material

Supplemental Materials (PDF)
