# Supplementary material for: The adjuvant GLA-SE promotes human Tfh cell expansion and emergence of public TCRβ clonotypes
Source: J Exp Med. 2019 Jun 7;216(8):1857–73. doi: 10.1084/jem.20190301 (PMC6683991; doi:10.1084/jem.20190301)
Supplement: Supplemental Materials (PDF) [file JEM_20190301_sm.pdf]

Supplemental material

Hill et al., https://doi.org/10.1084/jem.20190301

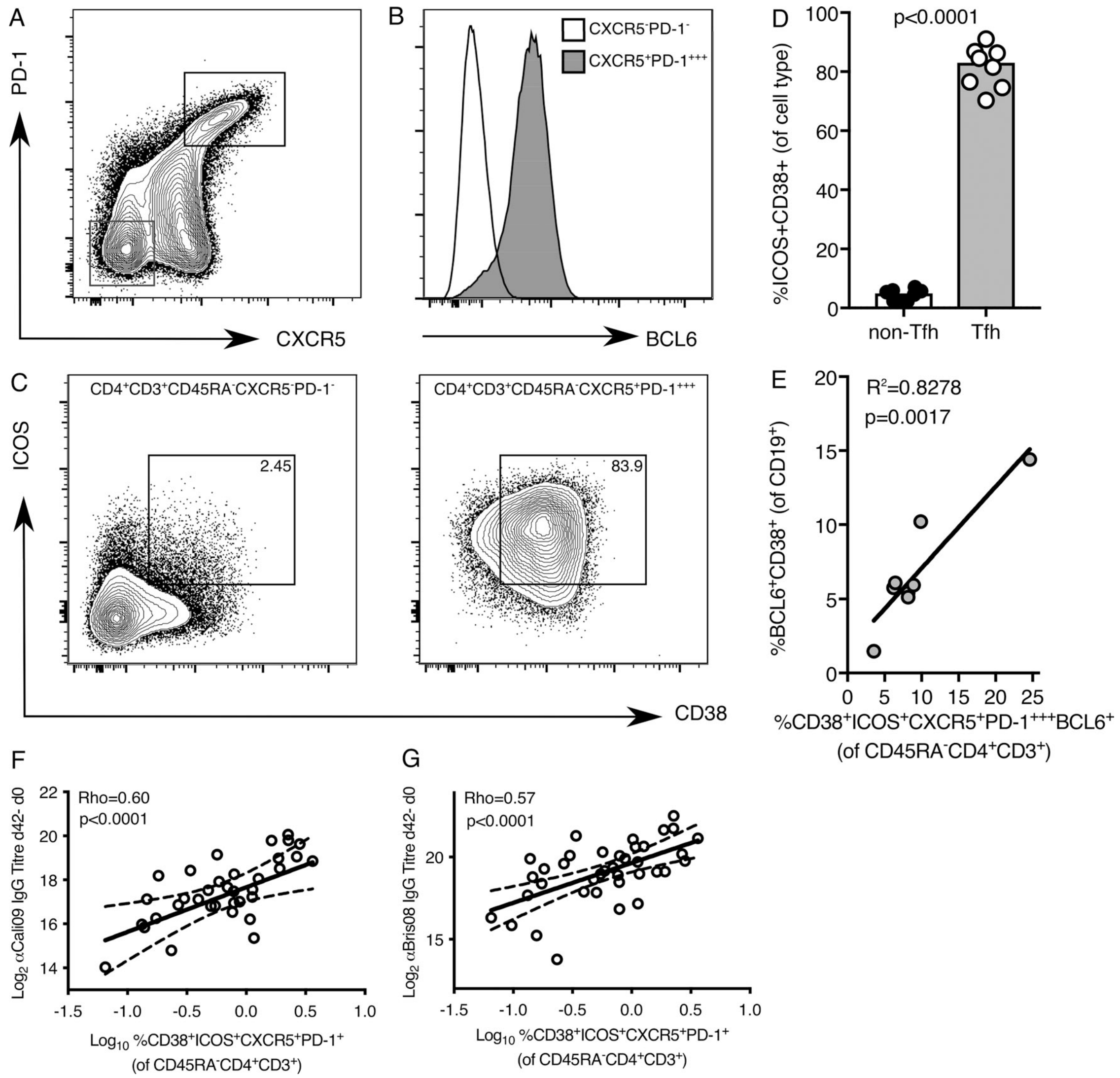

Figure S1. **Tonsil Tfh cells express CD38 and ICOS. (A and B)** Flow cytometric identification of (A) CXCR5$^+$PD-1^{+++} Tfh cells and CXCR5$^-$PD-1$^-$ non-Tfh cells among CD45RA$^-$CD4$^+$CD3$^+$ tonsil cells and their expression of BCL6 (B). **(C and D)** Contour plots (C) and quantification (D) of ICOS and CD38 expression on tonsil non-Tfh (left) and Tfh cells (right); *n* = 8. P < 0.0001. The P value was calculated by a Mann-Whitney test. **(E)** Linear regression analysis of tonsil BCL6$^+$CD38$^+$CD19$^+$ GC B cells and BCL6$^+$CD38$^+$ICOS$^+$CXCR5$^+$PD-1^{+++} Tfh cells; *n* = 7. R^2 = 0.8278; P = 0.0017. Each symbol represents one individual. Data are representative of one independent experiment. **(F and G)** Correlation of the frequency of CD38$^+$ICOS$^+$CXCR5$^+$PD-1$^+$ cTfh cells 7 d after vaccination with the change in antibody titer of anti-Cal09 IgG (F, an influenza A HA; Rho = 0.60; P < 0.0001) and anti-Bris08 IgG (G, an influenza B HA; Rho = 0.57; P < 0.0001) 42 d after vaccination. Statistical analysis by Spearman's correlation (Rho = coefficient); *n* = 41. F and G are from one seasonal influenza vaccination cohort.

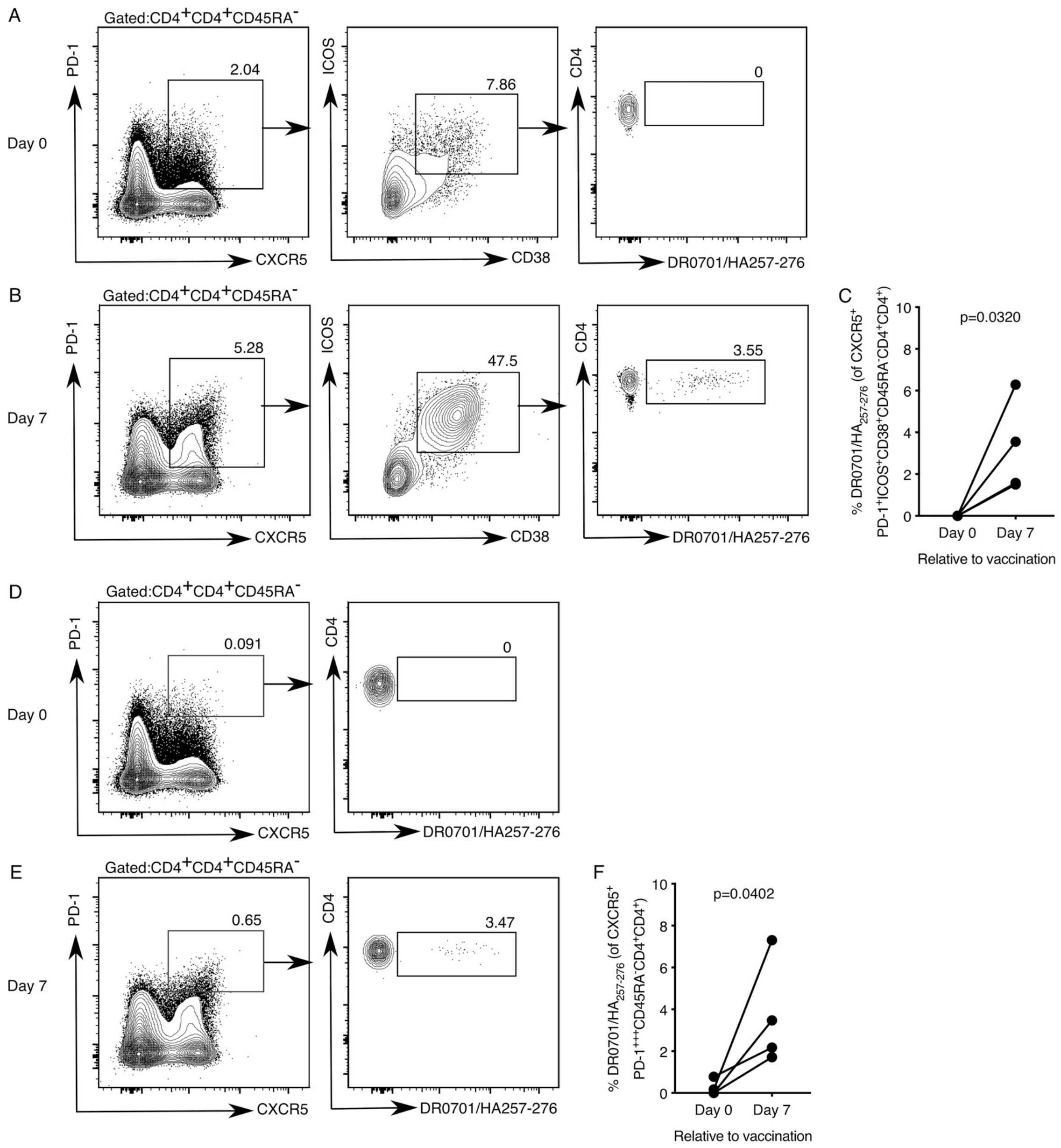

Figure S2. **HA-specific cTfh cells can be identified 7 d after seasonal influenza vaccination. (A–C)** Flow cytometric contour plots (A and B) and quantification (C) of the frequency of HLADR0701/HA$_{257\text{-}276}$ tetramer binding cells among CD38^{+}ICOS^{+}CXCR5^{+}PD-1^{+}CD45RA^{-}CD4^{+}CD3^{+} cells in the peripheral blood of healthy UK donors at days 0 and 7 relative to seasonal influenza vaccination; n = 4. In C, each symbol represents a volunteer; an individual donor is connected by a line at the two time points; n = 4. P = 0.0320. The P value was generated with a paired Student's t test. **(D–F)** Flow cytometric contour plots (D and E) and quantification (F) of the frequency of HLADR0701/HA$_{257\text{-}276}$ tetramer binding cells among CXCR5^{+}PD-1^{+++}CD45RA^{-}CD4^{+}CD3^{+} cells in the peripheral blood of healthy UK donors at days 0 and 7 relative to seasonal influenza vaccination; n = 4. In F, each symbol represents a volunteer; an individual donor is connected by a line at the two time points; n = 4. P = 0.0402. The P value was generated with a paired Student's t test. Data are from one seasonal influenza cohort.

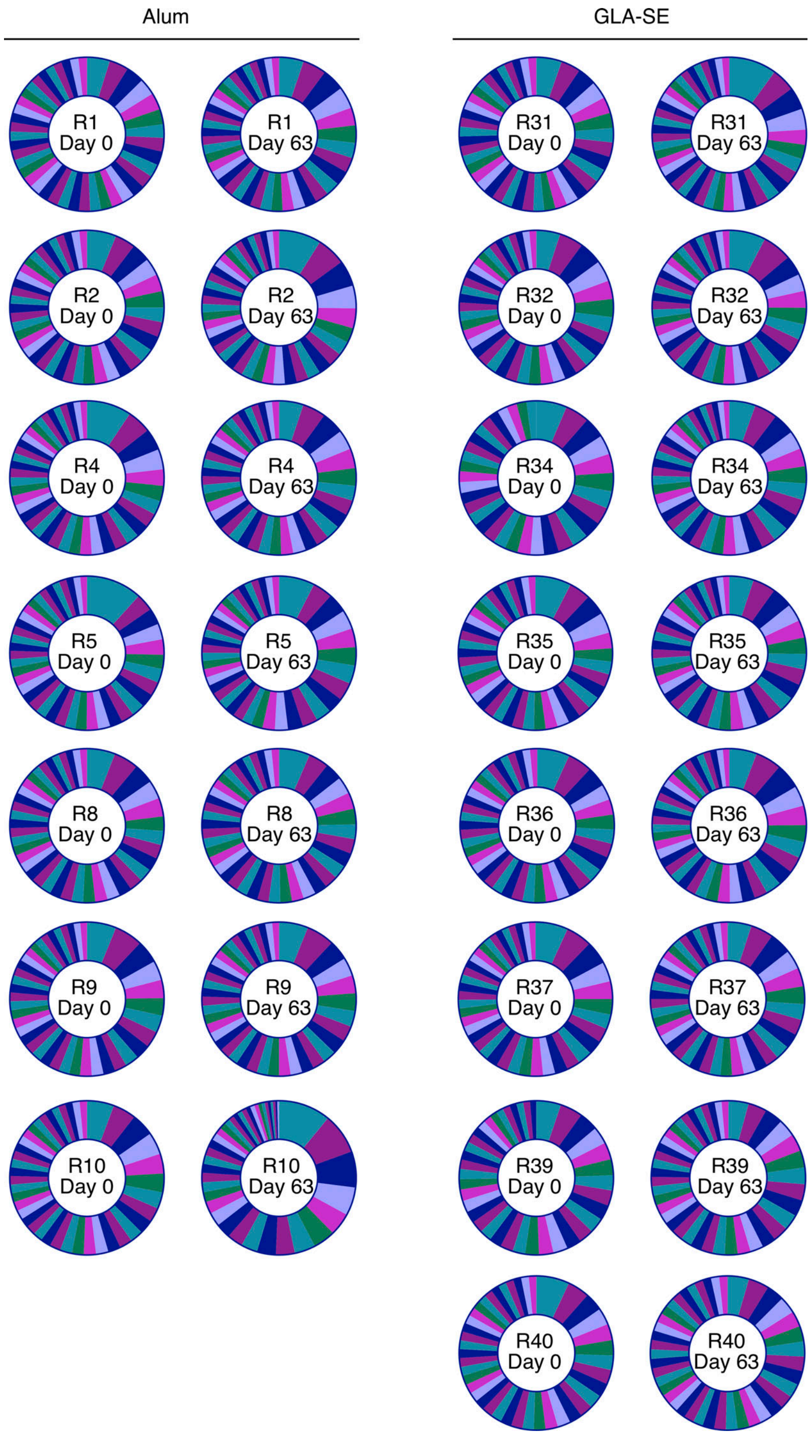

Figure S3. **Abundance of TCRβ CDR3 clonotypes in cTfh cells.** Pie charts of the proportion of the 40 most abundant TCRβ CDR3 clonotypes in ICOS$^+$CD38$^+$CXCR5$^+$PD-1$^+$ cTfh cells of individual P27A study participants at the indicated study time points. Alum group, *n* = 7; GLA-SE group, *n* = 8. Each segment of the pie chart represents a unique CDR3 clonotype. The "R" number is the unique participant identifier. Data are from one clinical trial.

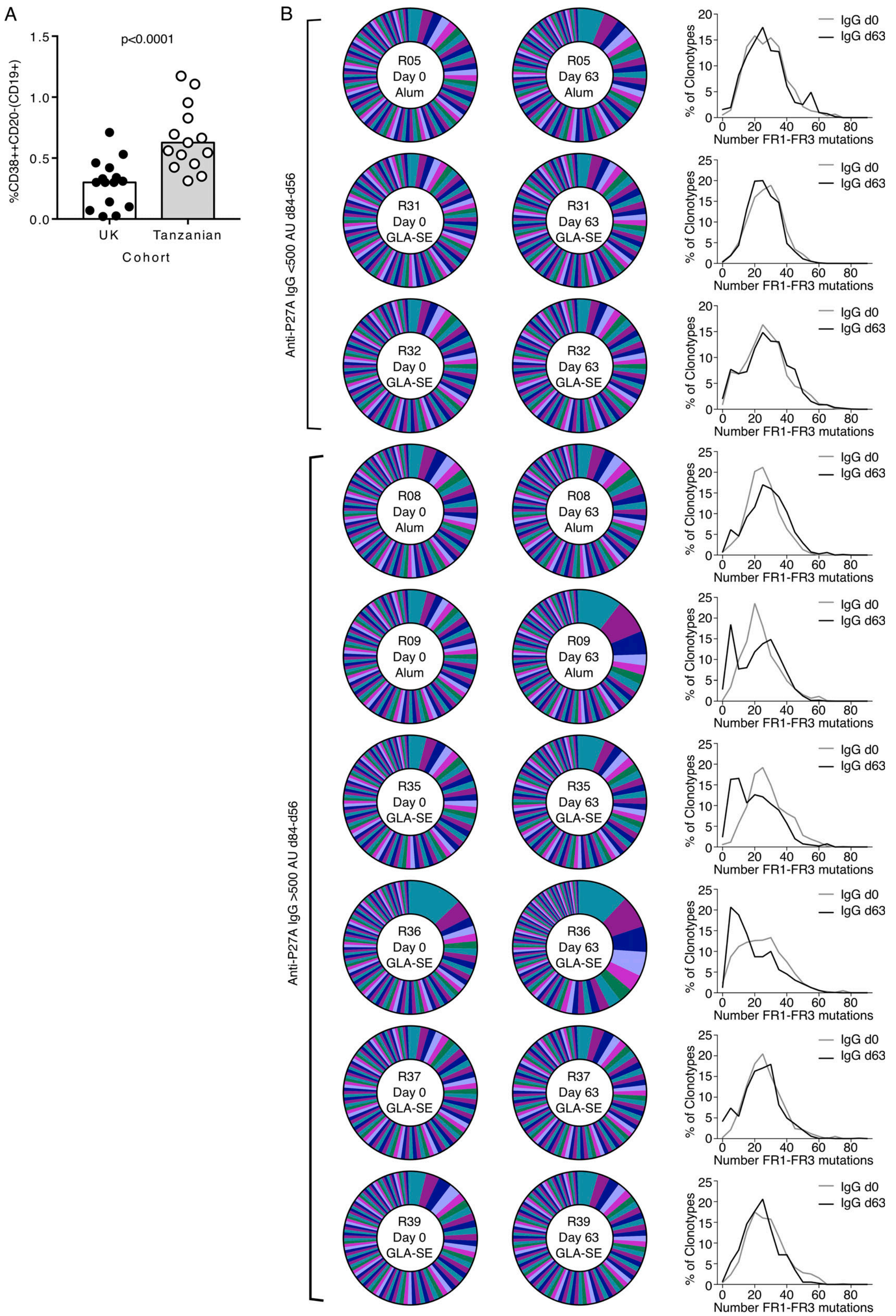

Figure S4. **Tanzanian volunteers had a higher ASC frequency before vaccination and V(D)J sequencing of circulating plasmablasts. (A)** Percentage of $CD19^+$ lymphocytes that are $CD38^{2+}CD20^-$ in 18–45-yr-olds from either the UK (*n* = 15) or Tanzania (*n* = 15) before vaccination. P < 0.0001. The P value was calculated from a Mann-Whitney *U* test. **(B)** Pie charts of the proportion of the 100 most abundant BCR clonotypes in $CD38^{2+}CD20^-CD19^+$ ASCs of individual P27A study participants at the indicated study time points. Each segment of the pie chart represents a unique BCR clonotype (FR1–FR4 regions). Alum group, *n* = 3; GLA-SE group, *n* = 6. The "R" number is the unique participant identifier. Line graphs show the number of mutations in the V region per clonotype (FR1–FR3, split into bins of five mutations) in each individual before vaccination and 7 d after the third vaccination (d63). Data are from one clinical trial.

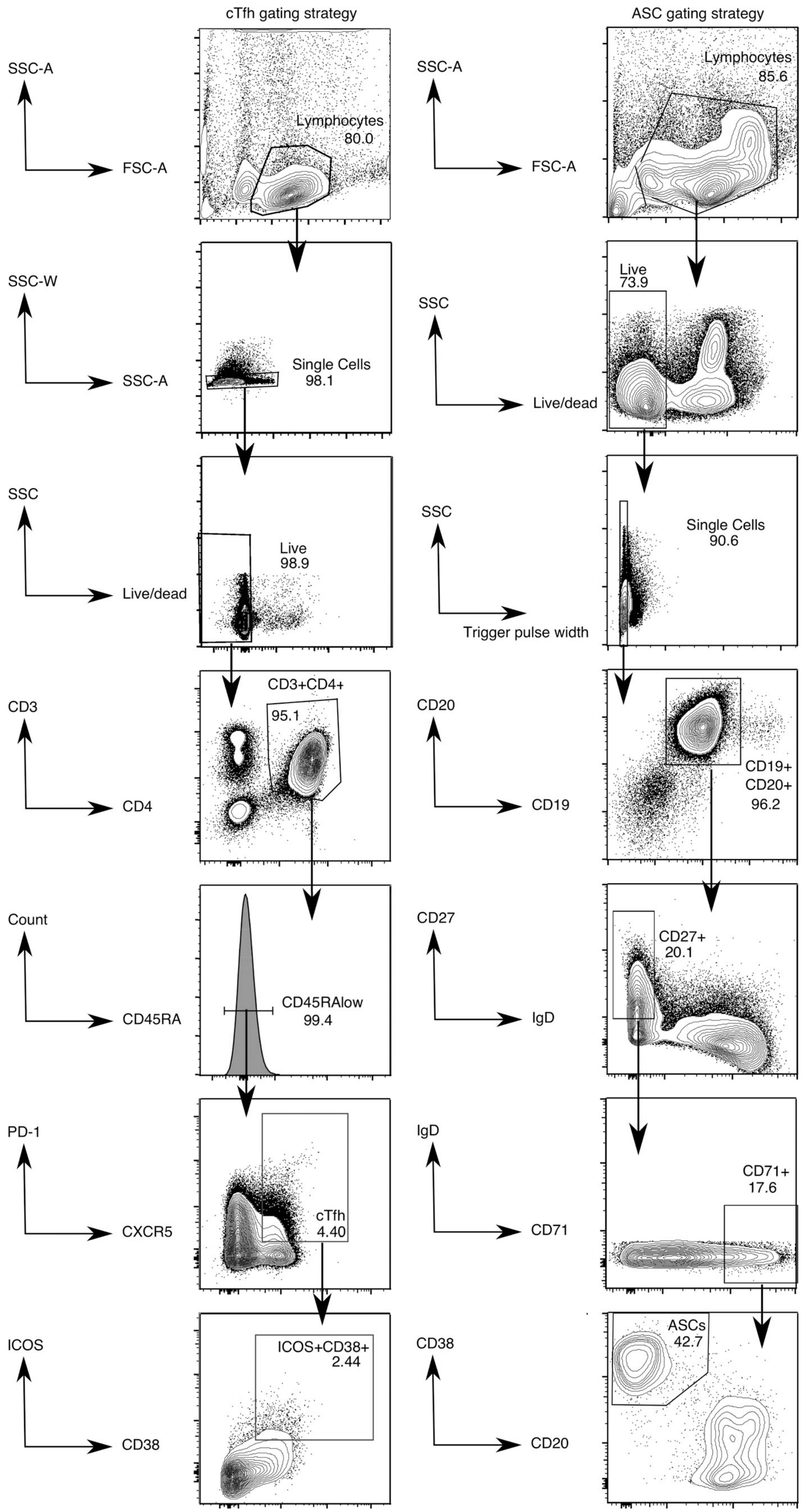

Figure S5. **Gating strategy for fluorescence-activated cell sorting of cTfh and ASCs.** Representative flow cytometry plots for the gating strategy for cTfh cells (left) and ASCs (right) with parameter axes labels shown alongside.
